# Peers for the fearless: Social norms facilitate preventive behaviour when individuals perceive low COVID-19 health risks

**DOI:** 10.1371/journal.pone.0260171

**Published:** 2021-12-09

**Authors:** Bernhard Kittel, Fabian Kalleitner, David W. Schiestl

**Affiliations:** Department of Economic Sociology, University of Vienna, Wien, Austria; University of Edinburgh, UNITED KINGDOM

## Abstract

A strategy frequently adopted to contain the COVID-19 pandemic involves three non-pharmaceutical interventions that depend on high levels of compliance in society: maintaining physical distance from others, minimizing social contacts, and wearing a face mask. These measures require substantial changes in established practices of social interaction, raising the question of which factors motivate individuals to comply with these preventive behaviours. Using Austrian panel survey data from April 2020 to April 2021, we show that perceived health risks, social norms, and trust in political institutions stimulate people to engage in preventive behaviour. A moderation analysis shows that the effectiveness of social norms in facilitating preventive behaviour increases when people’s perceptions of health risks decrease. No such moderation effect is observed for trust in political institutions. These results suggest that strong social norms play a crucial role in achieving high rates of preventive behaviour, especially when perceived levels of health risks are low.

## Introduction

SARS-CoV2, the severe acute respiratory syndrome coronavirus 2, has kept the world in suspense since it began to spread globally in early 2020. The pandemic prompted governments and civil societies to make enormous efforts to contain the spread of this virus. Until a substantial share of the population is vaccinated, “preventive behaviour”, which includes physical distancing, wearing a face mask, and minimizing social contacts, is considered the prime strategy to curb the spread of SARS-CoV2 [1]. While these preventive measures appear effective [2–7], their impact crucially depends on high rates of adoption in the population [8]. Given that preventive behaviour demands significant changes in practices of social interaction and lifestyles hitherto perceived as normal [9], individual adherence to these governmental measures is not guaranteed [10]. The resulting volatility in compliance threatens to undermine the governments ability to curb infection rates, which can lead them to implement stricter measures entailing great social and economic costs. This paper studies the factors influencing and stabilizing individual willingness to engage in preventive behaviour in different stages of the pandemic.

So far, the literature on preventive behaviour during the COVID-19 pandemic has mostly concentrated on the first few months of the pandemic in early 2020. We argue that this focus has potential drawbacks because developments during the pandemic not only fundamentally changed individual perceptions of the costs and benefits of preventive behaviour, but also changed the “contexts” in which individuals make behavioural decisions [11]. Different social contexts in form of fast-paced changes in government regulations and varying information about the current state of the pandemic influence citizen’s ability to deviate from publicly promoted preventive behaviour. This highlights the importance of the emergence and stability of new behavioural “norms” during the pandemic. Similar to Diekmann (2020) [12], who follows the seminal theory of Ullmann-Margalit on the emergence of norms [13], we argue that preventive behaviour constitutes a cooperation problem because individuals profit by deviating from the socially optimal behaviour, which potentially crowds out the norm-conforming behaviour of others [14]. When perceptions of health risk are high, individuals should engage in preventive behaviour without needing further incentives. When health risk perceptions are low, individuals may view preventive measures as comparatively costly, which increases the risk of free riding. Thus, low health risk perceptions should increase the strength of, and necessity for, further factors that can effectively guarantee widespread individual compliance with measures mandating preventive behaviour.

Using Austrian panel data, we study two social mechanisms affecting preventive behaviour and provide evidence of their relative strength depending on individually perceived levels of health risks. We show that, beyond health risks, perceived social norms and, to a lesser degree, trust in important governmental institutions are important factors promoting preventive behaviour. Moreover, in line with our expectations, the effect of social norms increases as individual health risk perceptions decrease. On the other hand, we find no substantive evidence that the effect of trust in institutions increases with decreasing levels of perceived health risks. These results highlight the importance of social networks and peer groups that provide information about the cooperation of others and the effectiveness of governmental measures. Most importantly, we show that social norms stabilize conditional cooperation in the form of preventive behaviour, in particular when individuals perceive minimal health risks.

In the following sections we provide a literature review of the main social factors that affect preventive behaviour and explain our argument that their explanatory power depends on health risk perceptions. Subsequently, we describe the data and our estimation strategy. After presenting some descriptive data about the development of the pandemic in Austria and our main variables of interest, we present the main findings estimating average marginal effects using two-way fixed effects models. Afterwards, we provide several robustness checks and discuss our findings in light of recent findings in the literature on COVID-19 and previous pandemics. We end by highlighting the potential importance of our findings for policies aiming to foster the uptake of preventive behaviour during pandemics.

## Primary predictors of preventive behaviour

To date, the literature on COVID-19 and previous pandemics has highlighted three main social mechanisms that facilitate preventive behaviour: people may believe in the existence of a threat and act in response to their health risk perception [15], they may adhere to a social norm [16], or they may act out of trust in the institutions responsible for containing the pandemic [17]. First, multiple studies of previous pandemics [18, 19] and of COVID-19 [20, 21] have shown that a *concern about health risks* can induce preventive behaviour [22] or increase intentions to accept a vaccine [23]. While there is still a debate about whether the strength of this effect depends on concerns about one’s own [24] or others’ vulnerability [25] to an infection, recent studies suggest that both factors encourage preventive behaviour [26].

Second, *social norms* refer to mutually expected behaviour [27, 28]. The literature distinguishes two main types of social norms: while “descriptive” norms refer to the observed behaviour of others, “injunctive” norms capture the expected moral approval of other people [29]. Jointly, both forms of social norms foster adherence to preventive behaviour in the context of COVID-19 [30, 31], as people may engage in preventive behaviour not because of their belief in the benefit of the behaviour itself, but because they care about their social relations and their reputation in the social environment [32–36]. In line with these expectations, empirical evidence regarding COVID-19 suggests that people who engage in preventive behaviour are perceived as more prosocial [37] and express less positive attitudes towards those not wearing masks [9]. Furthermore, people feel less “strange” wearing masks when amongst other people wearing masks [32] and individuals with friends in areas highly affected by the pandemic increase social distancing behaviour [38].

Third, *trust in authorities and legal measures* may promote human behaviour in line with government recommendations [39, 40]. Supporting these expectations, studies have shown that low levels of trust undermine a government’s ability to enact controversial policies [41] and decrease compliance [42]. Hence, trust in authorities and institutions is expected to facilitate compliance with measures to contain a pandemic, such as preventive behaviour [43]. In line with these arguments, initial empirical results regarding COVID-19 suggest that political trust indeed promotes preventive behaviour [17, 44–47].

## Theoretical background

We argue that, though these three factors represent distinct mechanisms, their effects on preventive behaviour are not independent of each other. The goal-framing theory developed in cognitive sociology [48] provides a useful theoretical framework for systematizing these key motivations for preventive behaviour. Central to this framework is the distinction between three layers of goals: “[…] the *hedonic* goal ‘to feel better right now,’ the *gain* goal ‘to guard and improve one’s resources,’ and the *normative* goal ‘to act appropriately.’ When such a goal is activated (i.e., when it is the ‘focal’ goal), it will influence what persons think of at the moment, what information they are sensitive to, what action alternatives they perceive, and how they will act” [49].

We use this perspective on agency theory, in which rationality is interpreted in terms of an interaction between self-regulation and social regulation, as an analytical framework for developing hypotheses on individual behavioural responses to the pandemic. The threat of COVID-19 can be understood as an exogenous shock that disrupts people’s hedonic routines and activates their self-regarding motives. Given that health is a priority issue for individuals [48], people who are concerned about their health should not need further inducement to implement measures to avoid infection. However, when people do not perceive the pandemic as a health risk, they may nevertheless engage in preventive behaviour because they believe it is appropriate, either for normative reasons [50, 51] or because they trust in the adequacy of policies developed by authorities [52, 53].

Previous empirical studies with respect to COVID-19 have reported puzzling results regarding the effects of social norms and political trust on preventive behaviour. While some studies, using data from the first surge of infections in early 2020, suggest that perceived health risks are the single most important factor in facilitating preventive behaviour [20, 21], other studies have highlighted the importance of other factors like political trust and social norms [9, 30–32]. We argue that accounting for the intervening factor of perceived health risks might explain these disparate findings, meaning that elevated perceptions of individual health risks (as reported in many countries at the beginning of the pandemic) should reduce the effects of trust and social norms on preventive behaviour because these social mechanisms are more relevant when health risk perceptions are low and thus do not induce people to take preventive measures out of self-interest.

This idea is also in line with the theory of normative social behaviour [54], which explicitly highlights the important role of outcome expectations for the explanatory power of social norms. According to this perspective, health risks are still crucial, but successful prevention strategies do not depend on high-risk perceptions. Empirical findings regarding health promotion have shown that perceived benefits moderate the effect of descriptive norms in promoting health-preserving behaviour [55]. In the context of COVID-19, studies have shown that the effects of social mechanisms facilitating preventive behaviour become stronger as levels of individually perceived health risks decrease [56]. Moreover, recent results from a field experiment in Bangladesh suggest that people’s aversion to a light informal social sanction is important for facilitating mask wearing over a longer period of time [57], even when masks have been distributed free of charge in that area. This again suggests that social norms are important facilitators of preventive behaviour in low-cost contexts. Therefore, the effects of social norms and trust in institutions should depend on the perception of health risks, that is, their effects should increase as the perception of health risks decreases.

## Research questions and hypotheses

These considerations lead to the following two questions: (Q1) What effects do perceived health risks, social norms, and trust in institutions exert on preventive behaviour to contain the SARS-CoV-2 pandemic? (Q2) How do immediate health concerns influence the effects of other social mechanisms on preventive behaviour?

Based on the theoretical arguments outlined above, we test three hypotheses (H1, H2, H3) corresponding to Q1 and two hypotheses (H4, H5) related to Q2:

(H1) The larger the individual concern about health risks, the higher is adherence to preventive behaviour.
(H2) The stronger individual perceptions of social norms of preventive behaviour, the higher is adherence to preventive behaviour.
(H3) The higher individual trust in institutions managing the pandemic, the higher is adherence to preventive behaviour.
(H4) The smaller the individual concern about health risks, the stronger is the effect of social norms on preventive behaviour.
(H5) The smaller the individual concern about health risks, the stronger is the effect of trust in institutions on preventive behaviour.

To test these hypotheses, we use panel survey data from a representative sample of the Austrian population. We analyse fixed-effects regression models of an index of preventive behaviour based on respondents’ self-reported likelihood to stay at home, wear masks, and keep physical distance from others. Together, these measures are considered essential individual contributions to governmental efforts to curb the pandemic, which may contribute to avoiding more severe measures such as the closing of infrastructure (commerce, schools) or regional or nationwide “lockdowns”.

## Methods

### Data

The data comprises eight waves (waves 3, 7, 11, 14, 16, 18, 20 and 22) of the *Austrian Corona Panel Project* (ACPP) [58], which includes questions on preventive behaviour and social norms. The observed period ranges from mid-April 2020 to mid-April 2021. This period includes parts of the first COVID-19 induced lockdown in Austria as well as the successive periods of relaxation and re-intensification of the pandemic and government measures in Austria. The ACPP is carrying out an online panel survey representative of the Austrian population with N = 1500, which is administered by a market research company. Details on the research design, panel attrition, as well as on the quota sampling that matches the Austrian population in terms of sociodemographic characteristics such as gender, age, education, employment status, migration background and region, are documented elsewhere [59]. For further information on data availability refer to (S1 Appendix D1 in S1 File). The panel survey is still ongoing, and we use the latest data containing the most relevant survey modules available at the time of conducting this study. However, results remain highly consistent even when we do not use the full sample available (see S1 Appendix B2 in S1 File).

### Measures

We operationalize the *dependent variable*, preventive behaviour, by means of a normalized additive index comprising three variables: (i) self-reported frequency of staying at home except for necessities, (ii) self-reported frequency of keeping a distance of at least one meter from others, and (iii) self-reported frequency of wearing a mask whenever physical distancing is not possible, with all items measured by a five-level Likert scale ranging from “almost always” to “almost never”. Cronbach’s alpha for this index is .69.

The *independent variables* we use to test our hypotheses comprise (1) perceptions of health risks coming from COVID-19, (2) social norms, and (3) trust in institutions. Again, we operationalize these three aspects through normalized additive indices based on 5-level (1,2) and 11-level (3) Likert scales:

1. The index of perceived health risks is based on respondents’ assessments of (i) the health risks COVID-19 entails for themselves and (ii) for the Austrian population in general. Cronbach’s alpha for this index is .76.
2. The index of social norms consists of (i) descriptive norms and (ii) injunctive norms regarding preventive behaviour. Descriptive norms refer to perceptions of other people’s behaviour, while injunctive norms refer to beliefs about other people’s opinions [60–63]. Thus, the items on injunctive norms asked respondents to “think of the *opinions of other people in Austria*. Please specify *how many* Austrians *hold the following opinions*.” The items on descriptive norms told respondents to think “of the *actual behaviour of other people in Austria*. From your perspective, please specify *how many* Austrians *engage in the following behaviour*” (emphasis in the original). Like the dependent variable, the social norms index includes items referring to descriptive and injunctive norms regarding staying at home (“They stay at home, except for necessary trips.”), keeping a distance (“In public, they keep a minimum distance of 1m from people who do not live in their household.”), and wearing a mask (“In public, they always wear protective masks.”). Cronbach’s alpha for this index is .82.
3. The index on trust in institutions consists of items on trust in four public institutions: (i) the government, (ii) the health care system, (iii) the parliament, and (iv) the police. Cronbach’s alpha for this index is .87. For all indices, the results of corresponding principal component analyses and the correlation of the relevant components to the additive indices used in the analyses can be found in S1 Appendix C1 in S1 File.

We control for the sociodemographic characteristics of gender, age, education, household size, migration background, and employment status. Employment status is also included in the FE-regressions and contains a dummy for flexible work arrangements (working from home), which has been recommended as a means of social distancing [64]. As the decision to work at home can be a deliberate attempt at preventive behaviour, we checked whether including this variable in our models decreased the other coefficient estimates. This was not the case (seeS1 Appendix B7 in S1 File). We recoded all variables in a way that aligns the direction and range of the scales (normalization), thus easing comparability. Hence, every variable ranges from 0 to 1, whereby 0 indicates the lowest and 1 the highest value of the corresponding concept (i.e. frequency, trust, agreement to statements, estimations of opinions and behaviour). The exact wording of all questions and the corresponding answer options in German, as well as their translation into English, can be found in S1 Appendix C2 in S1 File. We provide basic descriptive statistics for all variables used in the analyses in S1 Appendix A1 in S1 File.

A total of 2,631 individuals, including replacements for panel attrition, participated in the eight waves of the survey included in this analysis. Listwise deletion of missing values reduces the number of respondents to 2,408. Because we focus on variation within individuals, we further reduce the sample to respondents who participated in at least two of the aforementioned waves, which results in 2,030 individuals, providing a total of 10,210 observations. We provide balance checks on these samples in S1 Appendix B4 in S1 File. These results show that those who dropped out are on average more likely to have a migration background, to be female, younger, less educated and from larger households. However, with the exception of age, these effects are relatively small and inconsequential for the hypothesis tests. To avoid confounding by important socio-demographic characteristics like age (which may explain perceived health risks as well as preventive behaviour), we rely on within-individual variation in our main analyses. Crucially, those remaining in the sample are quite similar to those who dropped out with regard to the attitudes, perceptions and behaviours appearing in our main hypotheses. The only notable difference is that those who dropped out had, on average, lower levels of trust in institutions managing the crisis. 22 respondents (1.08%) reported a constant value on the preventive behaviour index over all waves in which they participated, thus providing no information for the fixed effects analysis.

### Analytic strategy

We use a two-way fixed-effects (2FE) panel model to avoid confounding by unobserved heterogeneity within individuals (refer to S1 Appendix D2 in S1 File for a link to the code used in this paper). Replacing individual fixed effects with time-invariant socio-demographic characteristics does not alter our substantive results for the variables of interest reported in the main analyses. This also holds true if we add further sociodemographic controls (see S1 Appendix A2 in S1 File, model 3). Because perceiving higher levels of social norms could potentially decrease respondents’ perceived health risks, we analysed whether this issue biases our estimates in S1 Appendix B8 in S1 File. We find that the medium-sized correlation between these variables decreases substantially when individual fixed effects are included, and that excluding perceived health risks from our models does not substantially change the effect of social norms on preventive behaviour. This suggests that the potential bias should be rather small. Furthermore, we include individuals’ perceived effectiveness of governmental measures in our models to control for some of the effect that social norms might have on preventive behaviour due to their correlation with risk perceptions. Again, this does not substantially change the estimates.

Because the dependent variable is censored at 0 and 1 by our normalization, we compare the linear approximation to a fractional probit model. We also compare our results to a tobit model because our estimates might be biased due to the truncation of the Likert scale measuring the three dimensions of preventive behaviour. However, both fractional probit and tobit models yield potentially inconsistent estimators in FE models, especially in unbalanced panels with small T [65, 66]. Thus, we report the results of standard linear two-way fixed effects models in our main analyses. However, we also provide regression estimates of both the tobit and the fractional model in S1 Appendix A2 in S1 File (models 6 and 7). These models do not yield substantially different results.

As suggested in the literature, we test whether the hypothesized moderations can be approximated by a linear interaction (see S1 Appendix B1 in S1 File) [67]. Wald tests provide p-values of 0.33 (social norms) and 0.04 (trust in institutions), suggesting that we should reject the NULL-hypothesis that, for the interaction of trust in institutions with perceived health risks, the point estimates of the binning estimators are statistically equivalent to linear interaction models. Hence, we also calculated the interactions without assuming linearity using kernel estimators. As these results are effectively similar to the linear model at important points in the distribution of the moderator (perceived health risks) and the explanatory variables of interest (perceived social norms and trust in institutions), we report the linear model in the main text and provide the results of binning as well as kernel estimators in the (S1 Appendix B1 in S1 File). Furthermore, we explore the plausibility of the parallel trends assumption, immanent in analyses using two-way fixed effects estimators [68]. We show that our results remain robust after the inclusion of leads (t+1) of the main variables, and that the effects only slightly decrease in size when we add individual specific wave trends (see S1 Appendix B3 in S1 File).

## Results

**Fig 1:**
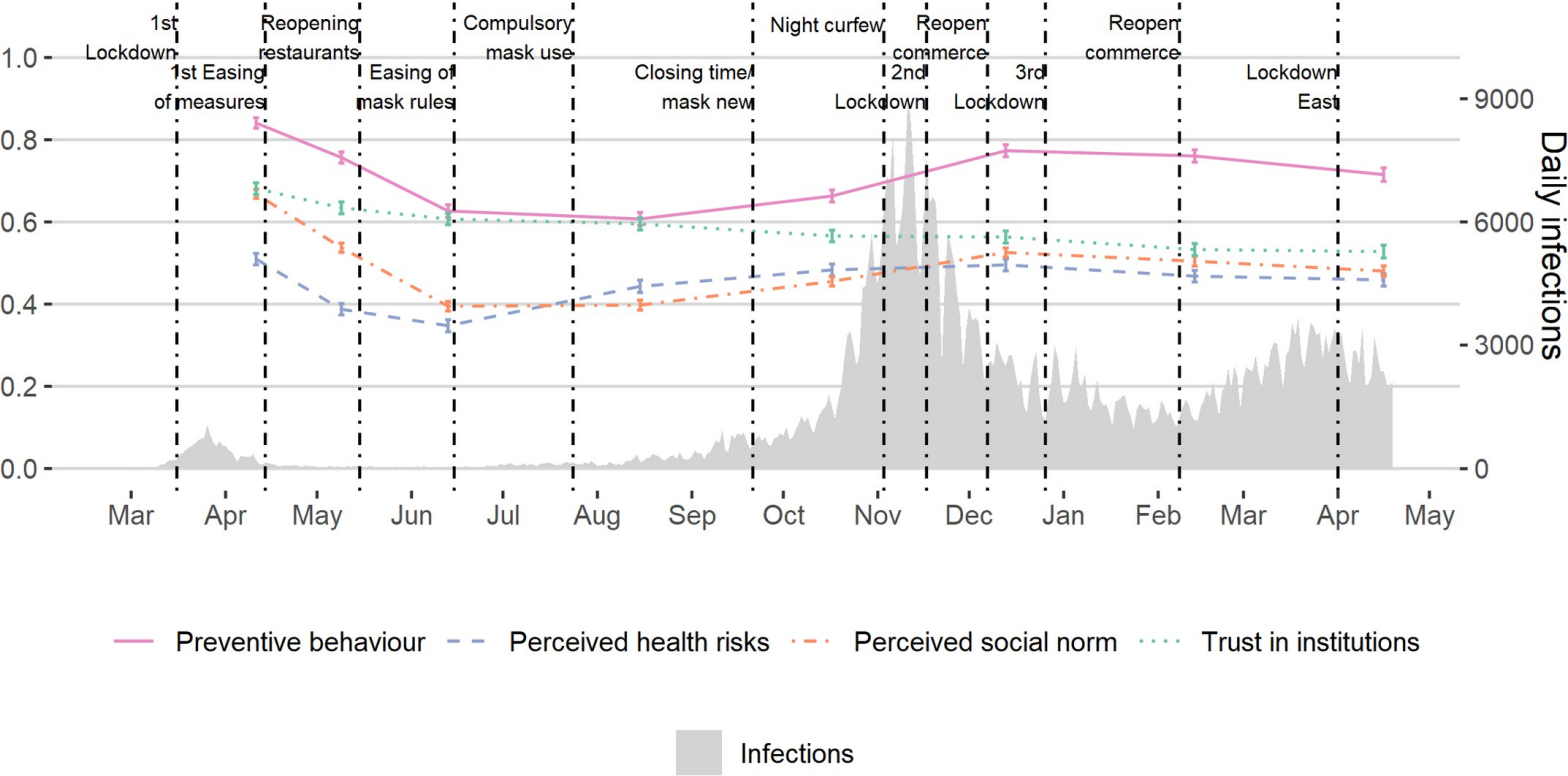
Evolution of preventive behaviour, perceived health risks, perceived social norms, trust in institutions, infections and government measures. Evolution of preventive behaviour, perceived health risks, perceived social norms and trust in institutions (N = 10210 obs. of 2030 individuals), along with official numbers of daily infections and date of introduction or relaxation of government measures. Each line displays the weighted values of corresponding normalized additive indices. Whiskers indicate 95% confidence intervals. See Methods for detailed descriptions of these variables and S1 Appendix in S1 File for descriptive statistics (A1) and question wordings (C2).

Fig 1 relates the evolution of preventive behaviour, perceptions of health risks, perceived social norms, and trust in institutions to the development of the pandemic over time. At the outset of the crisis, average adherence to preventive behaviour reached its maximum (.84; 95% CI [.83, .85]) to date on an index ranging 0–1 and then gradually declined following the reduction in the number of infections until it reached a minimum (.61; 95% CI [.59, .62]) in August 2020. Afterwards adherence increased again as the second, more severe, “wave” of COVID-19 infections hit Austria and the government (re)introduced strict measures to curb infection rates (December: .77; 95% CI [.76, .79]). In early 2021, adherence only decreased slightly as infection rates started to decrease and then picked up again (April: .72; 95% CI [.70, .73]). Similarly, average perceptions of health risks declined during spring 2020 (April: .51; 95% CI [.50, .52], June: .35; 95% CI [.33, .36]) and later rose in line with the incidence of infections, albeit at a slower rate than preventive behaviour (December: .50; 95% CI [.48, .51]). Also, the perception of a social norm of preventive behaviour, i.e. individual perceptions that others are adopting preventive behaviour and that they think this is the right thing to do, continuously declined during spring 2020 (April: .67; 95% CI [.66, .68], June: .39; 95% CI [.38, .41]). After remaining on a low level over the summer, it slightly increased in autumn and winter 2020 (December: .52; 95% CI [.51, .54]), but did not reach the earlier peak level despite the fact that similar governmental measures were in place. Trust in institutions also started at a high level (April: .68; 95% CI [.67, .70]) and exhibited a gradual decline during the crisis (June: .61; 95% CI [.59, .62]), but, unlike other indicators, it remained at low levels throughout the second half of 2020 and continued to decline in 2021 (April: 53; 95% CI [.51, .54]). Overall, the increase in infections from the end of the summer onwards did not trigger a behavioural response in the population as strongly as it did earlier in the pandemic. Even more so, however, people thought that others were not as strict in adopting preventive behaviours in the second and third waves of the pandemic compared to the first wave in 2020 despite much higher COVID-19 incidence rates.

**Fig 2:**
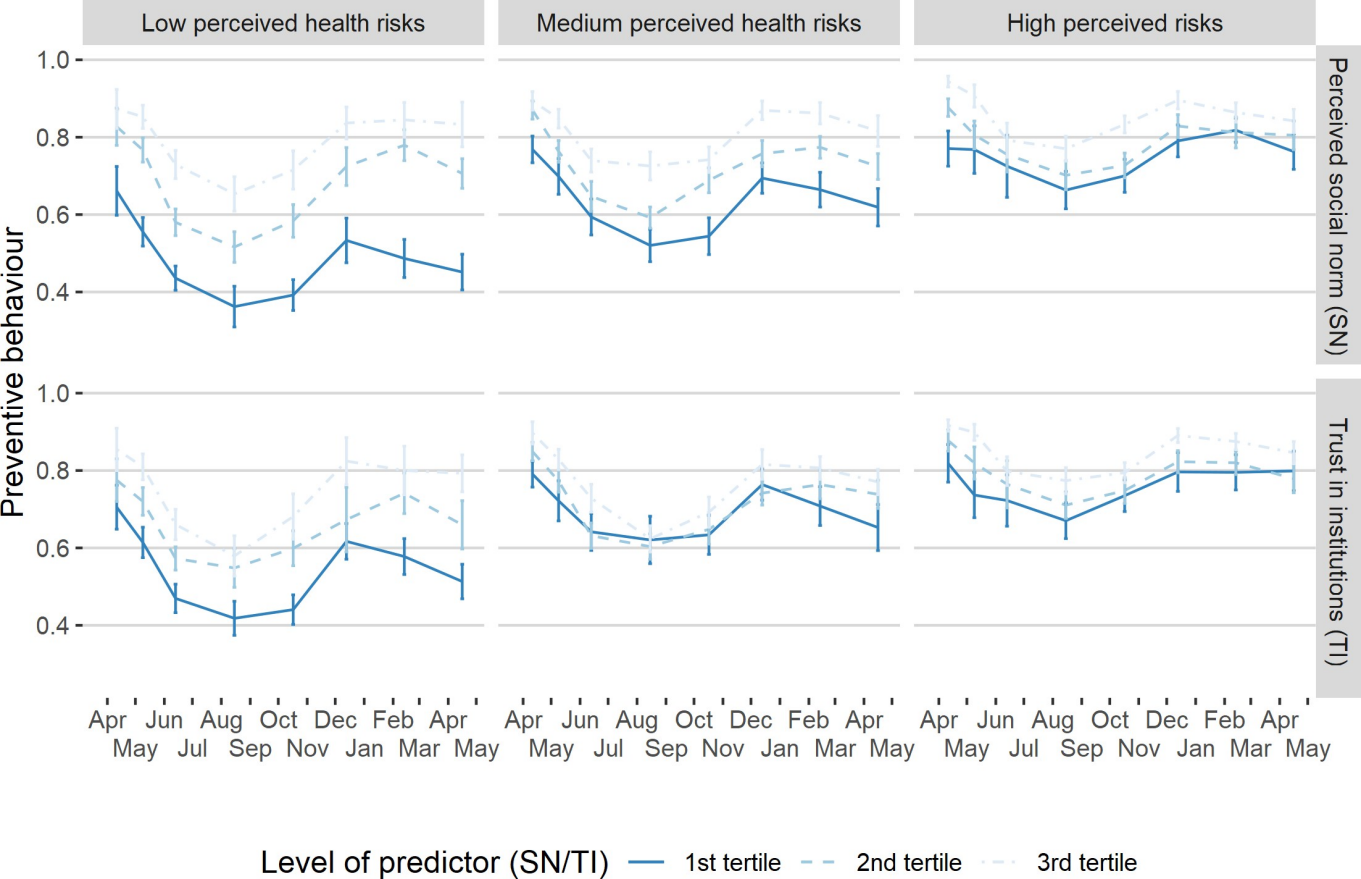
Evolution of preventive behaviour by different tertiles of perceived health risks, perceived social norm and trust in institutions. Evolution of preventive behaviour by different tertiles of perceived health risks, perceived social norm and trust in institutions (N = 10210 obs. of 2030 individuals). SN = Perceived social norms; TI = Trust in institutions. Whiskers indicate 95% confidence intervals. Levels of predictors (1^st^, 2^nd^, and 3^rd^ tertile) are calculated using within wave tertiles, each containing one third of our observations per wave. Low, medium and high perceived risk levels are calculated using overall perceived risk tertiles. See Methods for detailed descriptions of these variables.

While these observations indicate that, on average, perceptions of health risks, social norm perceptions and, to a lesser degree, trust in institutions changed in synchronicity, these variables are also cross-sectionally associated with preventive behaviour (Fig 2). Averaging over waves and respondents, we observe that respondents who perceive health risks to be high exhibit a value of .81 (95% CI [.80, .82]) on our 0–1 scale of preventive behaviour, compared to .61 (95% CI [.59, .62]) when they perceive the risks to be low (t = 37.1, p < .001, two-tailed). At the same time, preventive behaviour also increases with rising levels of respondents’ perceived social norms (high = .85 (95% CI [.84, .86]); low = .57 (95% CI [.55, .58]); t = 50.6, p < .001, two-tailed) and trust in institutions (high = .80 (95% CI [.79, .80]); low = .62, (95% CI [.61, .63]); t = 28.5, p < .001, two-tailed). However, the sizes of these associations substantially decrease when respondents perceive health risks to be high (social norm = .13 (95% CI [.11, .15]), trust in institution = .07 (95% CI [.06, .10])) compared to when they perceive them to be low (social norm = .34 (95% CI [.32, .36]), trust in institution = .18 (95% CI [.16, .20])).

To test whether these associations also hold when accounting for potential confounding factors due to changing conditions between waves (e.g. because of changing governmental measures), as well as to control for varying employment conditions (e.g. working from home), we next focus on the results of the linear regression models (see Methods). The baseline model (Model 1 in Table 1) tests the effects of perceived health risks, social norms and trust in institutions on preventive behaviour. It shows that an increase in perceived health risks corresponds to an increase in individual preventive behaviour, supporting H1. Similarly, the perception of social norms is positively associated with preventive behaviour in line with H2, as is trust in institutions in line with H3. However, the latter effect is substantially smaller in size. Model 2 includes interaction terms to test whether the effect of social norms and the effect of trust in institutions depend on the level of perceived health risks. All coefficients show effects in the expected direction, indicating higher effects of perceived social norms and trust in institutions when perceived health risks are low. Model 3 presents estimates from a two-way fixed effects panel regression that additionally accounts for unobserved heterogeneity that is constant within individuals in the observed period. Although the focus on within-individual variation decreases coefficient sizes, the estimates generally support the results of the OLS with wave fixed effects. However, the interaction between trust and perceived risk now fails to reach statistically significant levels. Model 4 additionally controls for the local infection rate, which might explain risk perceptions as well as the adoption of preventive behaviour, by including the 7-day incidence rate on the regional level (see Methods). Estimates in Table 1 indicate that this macro-level indicator is statistically insignificant, marginal in size, and has nearly no effect on the coefficients of the other variables in the model (β = .003, t = 1.34, p = .223; 95% CI [-.003, .009]. The coefficient implies that a tenfold increase in the regional incidence rate would increase preventive behaviour by less than .01). To interpret the significance and assess the strength and relevance of the estimated effects of social norms and trust in institutions on preventive behaviour at different levels of individual perceived health risks, we next focus on marginal effects plots [69].

**Table 1:** Preventive behaviour: OLS regression estimates.

	(1)	(2)	(3)	(4)
	Preventive behaviour	Preventive behaviour	Preventive behaviour	Preventive behaviour
Perceived health risks	0.269***	0.689***	0.385***	0.382***
	(0.0244)	(0.0425)	(0.0651)	(0.0637)
Perceived social norm	0.434***	0.763***	0.608***	0.610***
	(0.0166)	(0.0437)	(0.0364)	(0.0332)
Trust in institutions	0.121***	0.173**	0.107^+^	0.106^+^
	(0.0173)	(0.0342)	(0.0509)	(0.0481)
Perceived social norm X		-0.721***	-0.464***	-0.468***
Perceived health risks		(0.0769)	(0.0535)	(0.0436)
Trust in institutions X		-0.147*	-0.119	-0.112
Perceived health risks		(0.0565)	(0.0800)	(0.0772)
log(Regional 7day-incidence)				0.00339
				(0.00254)
Wave FE	Yes	Yes	Yes	Yes
Individual FE	No	No	Yes	Yes
Observations	10210	10210	10210	10020
Individuals	2030	2030	2030	1983

Table 1: Preventive behaviour: OLS regression estimates. Controls for changes in employment situation, as well as perceived effectiveness of measures. Robust standard errors in parentheses are clustered by wave and individuals (Models 1–3) or by wave, individuals, and region (Model 4) (* p < .05; ** p < .01; *** p < .001). The estimates are robust against the inclusion of further controls, alternative specifications of the link function (fractional model), and alternative assumptions about the data structure (tobit). For details refer to Methods. Full estimates are provided in (S1 Appendix A2 in S1 File).

**Fig 3:**
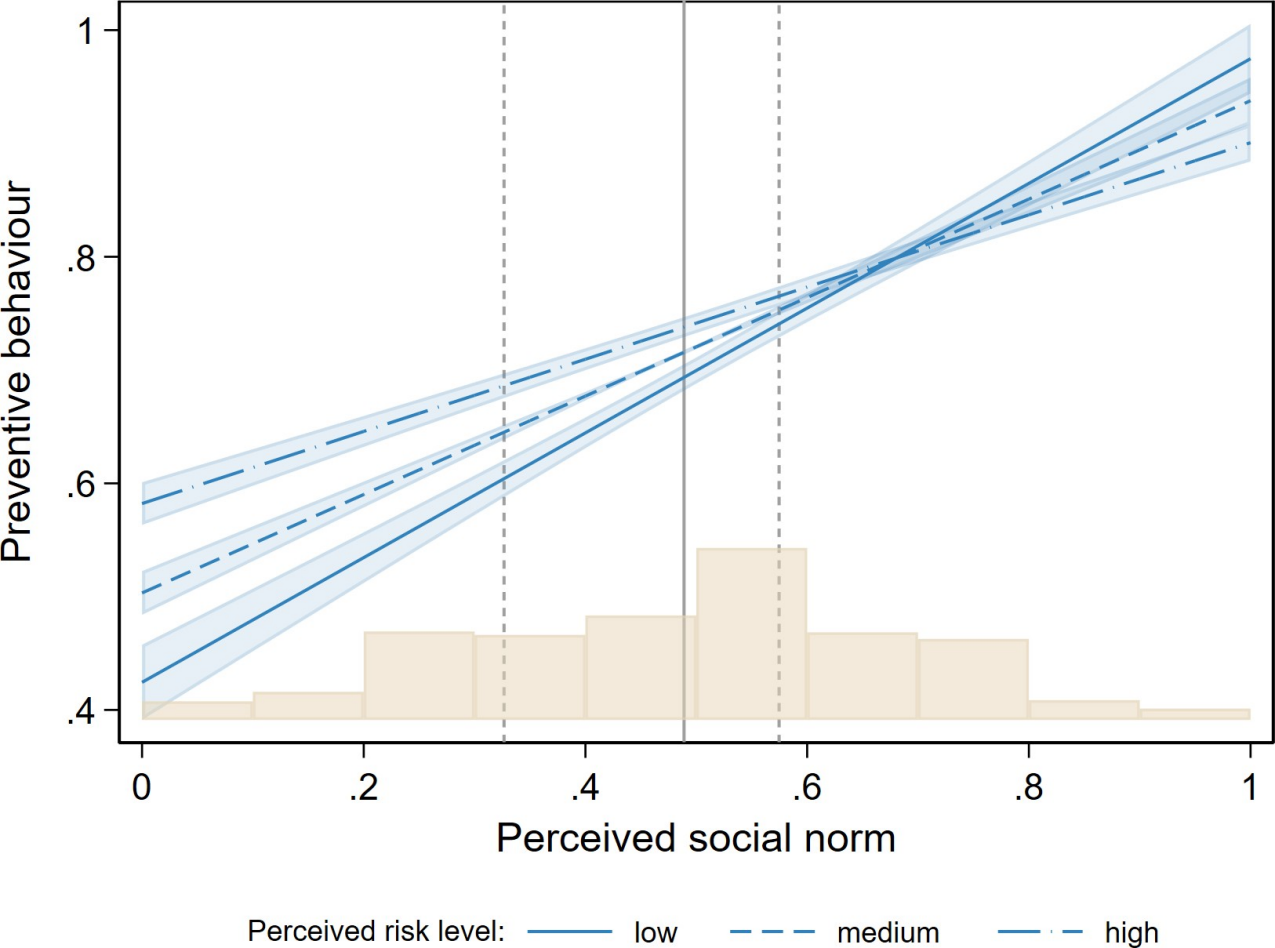
Linear prediction of preventive behaviour by perceived social norm conditional on the level of perceived health risks. Linear prediction of preventive behaviour by perceived social norm conditional on the level of perceived health risks. Predictions based on estimates in model 3 in Table 1. Linear Fixed Effects model with wave as well as individual fixed effects. 95% confidence intervals (light blue areas) are calculated using two-way clustered standard errors for individuals and waves. The histogram represents the distribution of social norm perceptions in the sample. Dashed lines mark a +/- one within-individual SD from the mean.

Fig 3 shows the effect of social norm perceptions on preventive behaviour for low, medium, and high levels of health risk perceptions (values of low, medium, and high health risk perception correspond to the 15, 47, and 86 percentiles, respectively). Holding other variables at their means and focusing on a medium level of risk, a one within-individual standard deviation increase in the perceived level of social norms from the mean is associated with a .05 (z = 22.22, p < .001; 95% CI [.05, .06]) increase in preventive behaviour. Relative to the observed within-individual standard deviation in preventive behaviour (henceforth called SD), this amounts to a .38 SD increase (z = 22.22, p < .001; 95% CI [.35, .41]), which is a moderately strong effect. Translated into our substantive measures this means that an increase in average perceived norms from thinking that “some people engage in preventive behaviour” to thinking that “most people engage in preventive behaviour” results in an increase of preventive behaviour by .25 (0 = almost never practice these behaviours, 1 = almost always practice these behaviours).

As Fig 3 shows, the size of the social norm effect (indicated by the gradient of the lines) depends on the level of individually perceived health risks. While the effect of perceived health risks is comparatively small (.08 SD at the mean; z = 4.31, p < .001; 95% CI [.04, .11]; when the average perceived risk increases from “low” to “high” our preventive behaviour index increases by .04), the perceived level of health risk also moderates the size of the effect of perceived social norms. Focusing on empirically observed points that have a high probability of occurring in the distribution of perceived health risk in our sample, we observe effect sizes of .43 SD (z = 20.81, p < .001; 95% CI [.39, .47]) and .32 SD (z = 21.53, p < .001; 95% CI [.29, .35]), respectively, for one standard deviation below and above the mean. Hence, the effect size of social norms decreases by .05 SD for a one SD increase in perceived health risks or, in relative terms, decreases by 25% when individually perceived health risks shift from moderately low to moderately high levels. This difference in the average marginal effects (AMEs) is highly significant according to a Wald test (-.11 SD, p < .001, 95% CI [-.13, -.08]), which provides strong evidence that smaller individual concerns about health risks imply a larger effect of social norms on preventive behaviour, supporting H4. Moreover, Fig 3 also indicates that at high levels of perceived social norms an increase in health risk perceptions does not substantially alter preventive behaviour.

**Fig 4:**
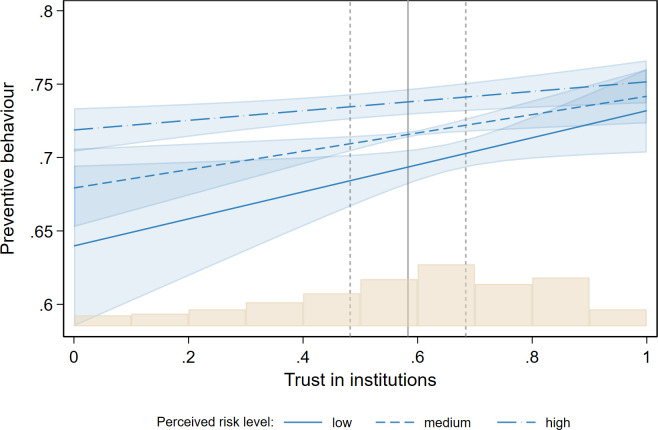
2FE linear regression linear prediction of preventive behaviour by trust in institutions conditional on the level of perceived health risks. 2FE linear regression linear prediction of preventive behaviour by trust in institutions conditional on the level of perceived health risks. Predictions based on estimates in model 3 in Table 1. Linear Fixed Effects model with wave as well as individual fixed effects. 95% confidence intervals (light blue areas) are calculated using two-way clustered standard errors for individuals and waves. The histogram represents the distribution of social norm perceptions in the sample. Dashed lines mark +/- one within-individual SD from the mean.

Fig 4 shows that, similar to the results on social norms, a rise in the level of trust in institutions increases preventive behaviour. Holding other variables at their means, a one standard deviation increase from the mean in trust in institutions is associated with a .04 SD (z = 2.95, p = .003; 95% CI [.01, .06]) increase in preventive behaviour. Thus, while statistically significant, the positive effect of trust on preventive behaviour at mean levels of perceived health risks is comparatively small. When average individual trust in institutions increases from moderately low trust (3/10) to moderately high levels of trust (7/10), preventive behaviour increases by only .02. This result is not only due to the smaller size of the coefficients but also because trust in institutions tends to be more stable within individuals than social norms and health risk perceptions (.10 compared to .13 for social norms and .12 for perceived health risks). Focusing again on relevant points in the distribution of perceived health risks, i.e. one standard deviation below and above the mean, effect sizes are .05 SD (z = 2.59, p = .010; 95% CI [.01, .09]) and .03 SD (z = 3.05, p = .002; 95% CI [.01, .05]), respectively. Hence the size of the effect of trust in institutions on preventive behaviour decreases by .01 SD for a one SD increase in perceived health risks. This difference in the AMEs, however, is not statistically significant according to a Wald test (-.02 SD, p = .137, 95% CI [-.05, .01]) (also refer to S1 Appendix B1 in S1 File for marginal effects plots of the interaction using kernel estimators to account for potential non-linearities in this moderation). Thus, the evidence does not support H5 that smaller perceived health risks lead to trust in institutions having a larger effect on preventive behaviour.

## Robustness checks

Besides health risks, social norms, and trust, previous studies have highlighted several other factors affecting preventive behaviour. Empirical results from the US indicate that health risk perceptions may be biased by selective consumption and reception of media reports, as suggested by the theory of political reasoning [70]. These risk perceptions in turn affect preventive behaviour. Furthermore, endorsement of preventive behaviour can depend on personality traits such as psychological entitlement [71], agreeableness, and conscientiousness [72]. As our study relies on within-individual variation, factors such as party affiliation and relatively stable long-term personality traits should not bias our estimates. However, we also tested whether accounting for party affiliation changes the estimates reported in Model 2 in Table 1. We found no effects on preventive behaviour, and including party affiliation did not substantially change the coefficients of our main variables (see S1 Appendix A2 in S1 File).

Another factor frequently highlighted in the literature is self-efficacy: people are more likely to behave in accordance with measures if they assume effectiveness [73] and performability [74]. This has also been shown in the context of COVID-19 [56]. To account for the fact that different beliefs about the effectiveness of COVID-19 measures might confound our estimates, we control for individuals’ perceived effectiveness of measures in general. These remain insignificant in all our analyses (see S1 Appendix A2 in S1 File). However, as this captures only one aspect of the concept of self-efficacy, we cannot exclude the possibility that other dimensions of self-efficacy may affect preventive behaviour.

To check the robustness of our results against alternative specifications of preventive behaviour, we test whether our main variables would explain nonessential mobility during the pandemic (meeting friends, going outside because of boredom). Our results remain robust when using this variable (see S1 Appendix B6 in S1 File). In a similar approach, we also apply a placebo check by testing whether our main variables of interest would fail to explain essential mobility (buying groceries or medicine, visiting a doctor). As expected, our main variables of interest fail statistical significance tests in this case (see S1 Appendix B6 in S1 File). This implies that our results capture the specific link connecting risks, trust, and social norms with preventive behaviours.

Different kinds of preventive behaviour might constitute different kinds of behavioural dilemmas. While avoiding unnecessary mobility always protects oneself and others, the health benefits of physical distancing and protective masks can be complex. To test whether the use of separate dimensions instead of an index of preventive behaviour would alter our results, we provide regression estimates using the different dimensions of each of the variables underlying the index of preventive behaviour as dependent variables in S1 Appendix A3 in S1 File. These results indicate that there are no substantial differences in direction or size of the effects for our main independent variables compared to the index of preventive behaviour. This provides evidence that, although some characteristics differ, people tend to view different kinds of preventive behaviour in the same light. For instance, while studies suggest that mask wearing is more effective in protecting others than oneself [6, 75], this was not common knowledge at the start of the pandemic. In addition, the Austrian government advertised the (later compulsory) FFP2 masks as a tool to protect others *as well as* oneself. Hence, people had reasons to believe that masks create health benefits for oneself and others.

The ACPP dataset consists of individual-level data, which enables nuanced studies that go beyond the aggregate analysis of behavioural change during the COVID-19 crisis [76, 77]. It allows for tests of individual-level mechanisms, thus avoiding ecological fallacies. However, because we cannot directly observe behaviour in a survey, we have to rely on self-reported behaviour. While this represents a limitation of our study, our estimation approach reduces the potential of biases in the results. Since we rely on within-individual variation to test the hypotheses, using self-reported data instead of data on actual behaviour only biases our estimates if changes in individual reported behaviour do not relate to changes in actual behaviour. Thus, utilizing variation within individuals over time should minimize the impact of social desirability bias. Moreover, recent empirical studies suggest that estimates of compliance with COVID-19 regulations do not suffer from social desirability tendencies [78, 79]. To test whether our data reflects macro-level behavioural changes in Austria, we compare the propensity of staying at home, which is the dimension of our preventive behaviour measure that most closely measures actual mobility, with macro data of mobility patterns provided by Google [80]. We find that our estimates follow average patterns quite well (see S1 Appendix B5 in S1 File). Moreover, we validate these results with other sources that provide aggregated mobility estimates in Austria [81] and again find patterns of change over time similar to our survey estimates. This corroborates results from another study that uses micro data and finds that the reported times people spend outside seem to be externally valid when checked against mobile phone data [82].

## Discussion

We used individual-level panel data from Austria spanning nearly the full duration of the COVID-19 pandemic thus far to analyse three factors promoting preventive behaviour: perceived health risks, social norms, and trust in institutions. We found that, on average, people with lower health risk perceptions, those who perceive less of a social norm of preventive behaviour among others, and those who have lower levels of trust in institutions responsible for dealing with the crisis, are less likely to adopt preventive behaviour. Moreover, we found that these effects are not independent from each other: a decline in the level of perceived health risks increases the relevance of social norms in facilitating preventive behaviour. We do not find robust evidence that changes in perceived health risks have a similar moderating effect on the relationship between trust in institutions and preventive behaviour. Fixed-effects regressions focusing on variation within individuals suggest a small effect of perceived health risks (.08 within-individual standard deviation increase in preventive behaviour after a one within-individual standard deviation increase in health risks), a moderately strong effect of social norms (.38), and a small effect of trust in institutions (.04) on preventive behaviour. Moreover, the effect of social norms decreases by 25% when risk perceptions increase from moderately low to moderately high levels (one within-individual standard deviation below and above the mean). These results are robust against alternative specifications of the link function and against alternative assumptions about the data structure (see Robustness Checks and Methods).

Our study applied a theoretical framework that highlights the mutually reinforcing nature of human behaviour [48], which is a crucial element of preventive behaviour in public health contexts. This approach recognizes that, in most cases, preventive behaviour mainly benefits others [20, 83–85], while its costs are primarily borne by the individual. In line with the results of previous studies on norm-violating behaviour [86] and the theory of normative social behaviour [54], our results highlight the potential of social norms to overcome the public goods dilemma inherent in preventive behaviour: in a positive feedback loop, an uptake of preventive behaviour induces similar behaviour among others. This result relates to expectations of conditional cooperation [87, 88] and research that highlights people’s concern about their reputation [36].

Our finding that perceived health risks function as an important moderator of other factors facilitating preventive behaviour also raises implications for policymaking and may help to explain the varying results of previous research on health behaviour: the conditional effects of social norms may explain the inconsistent results regarding the benefits of health messages in facilitating preventive behaviour during the COVID-19 pandemic [89–93]. While interventions aimed at raising individual risk perceptions may boost preventive behaviour in the short term, they may also lower the impact of other measures aimed at fostering preventive behaviour in the long term. This is especially relevant as the effect of social norms exceeds the effect of health risk perceptions: our results show that people who perceive high health risks but observe low compliance with preventive behaviour among others are still less likely to adhere to these norms themselves. Thus, lower degrees of perceived social norms can undermine individual willingness to comply even when individuals are highly concerned about potential health risks.

While our data is limited to the national context of Austria, our study has distinct advantages over some international comparative studies used in the literature. First, it does not rely on convenience samples, which are often used to get fast and easy access to respondents in a variety of countries. Second, the use of panel data over an extended period of time allows us to focus on variation within individuals and hence render irrelevant constant characteristics that may confound the results. Because of the panel structure, we are also able to test the effects of our main variables of interest at different stages of the pandemic, while controlling for changes in nationwide governmental regulations through wave fixed effects. Furthermore, the available literature so far has shown that risk perception correlates with health behaviour in a wide range of countries [94] and that national contexts do not substantially change the effects of individually perceived knowledge efficacy, interpersonal trust, and trust in institutions on preventive behaviour [56].

While focusing on within individual changes has several methodological advantages, this model also constitutes a limitation of the study. Besides variables that change during the pandemic, stable individual characteristics may influence health risk perceptions, social norm perceptions, and preventive behaviour. For instance, a rich literature in psychology suggests that personality traits (such as agreeableness) influence preventive behaviour and how closely people adhere to social conventions regarding healthy behaviour [11, 95, 96]. After accounting for time-invariant differences between individuals, this effect should be rather small in our case, but it may still be important to understand the stable differences between individuals in their adherence to preventive behaviour. We therefore argue that more research is needed to test the influence of these stable characteristics on the context-dependent effects of social norms highlighted here.

In view of the importance of preventive behaviour for curbing infection rates and the high demands it places on individuals to change common practices of social interaction, it is crucial to provide evidence on the heterogeneous factors that promote preventive behaviour [10, 26]. The results of this study indicate that high perceptions of social norms render low perceptions of health risks irrelevant, implying that social norms might function as an important lever for facilitating preventive behaviour. Thus, at the societal level, institutions responsible for dealing with the crisis need to maintain and build support. Transparent communication about expected behaviour [97, 98], exemplary behaviour by officials [99], and information campaigns can foster citizens’ uptake of the desired behaviour, as recent research on vaccine hesitancy suggests [100]. Furthermore, at the individual level, role models exhibiting compliant behaviour can support the development of social norms and facilitate compliance with pandemic response strategies [82, 101].

## Supporting information

S1 File Appendix. Online supplementary appendix.
(DOCX)
