# Supplementary material for: Peers for the fearless: Social norms facilitate preventive behaviour when individuals perceive low COVID-19 health risks
Source: PLoS One. 2021 Dec 9;16(12):e0260171. doi: 10.1371/journal.pone.0260171 (PMC8659684; doi:10.1371/journal.pone.0260171)
Supplement: S1 File — Online supplementary appendix. (DOCX) [file pone.0260171.s001.docx]

Peers for the Fearless: Social norms facilitate preventive behaviour when individuals perceive low COVID-19 health risks

S1 Appendix

[S1 Appendix A. Summary statistics and full regression estimates 2](#_Toc84325686)

[A1. Summary statistics 2](#_Toc84325687)

[A2. Full regression estimates 4](#_Toc84325688)

[A3. Single regressions: aspects of preventive behaviour (staying home, wearing masks, keeping distance) 5](#_Toc84325689)

[S1 Appendix B. Checks of model assumptions and robustness of the moderation 6](#_Toc84325690)

[B1. Test linearity of moderation effects 6](#_Toc84325691)

[B2. Check robustness of moderation against sample restrictions 9](#_Toc84325692)

[B3. Check of parallel trends assumption 11](#_Toc84325693)

[B4. Balance checks of data 12](#_Toc84325694)

[B5. Comparison of survey and mobile data (external validity) 13](#_Toc84325695)

[B6. Alternative operationalization of preventive behaviour and placebo check 14](#_Toc84325696)

[B7. Testing the influence of home-office 15](#_Toc84325697)

[B8. Assessing multicollinearity between perceived social norms and perceived health risks and testing for potential mediating effects 16](#_Toc84325698)

[S1 Appendix C. Variables, indices, and question wordings 18](#_Toc84325699)

[C1. Variables and indices 18](#_Toc84325700)

[C2. Questions and answer options 24](#_Toc84325701)

[S1 Appendix D. Data and Code Availability 30](#_Toc84325702)

[D1. Data Availability 30](#_Toc84325703)

[D2. Code Availability 30](#_Toc84325704)

[S1 Appendix References 31](#_Toc84325705)

## S1 Appendix A. Summary statistics and full regression estimates

### A1. Summary statistics

| Variable | N | Mean | SD | Min | Max |
| --- | --- | --- | --- | --- | --- |
|  |  |  |  |  |  |
| Preventive behaviour | 10210 | 0.72 | 0.24 | 0 | 1 |
| Perceived health risks | 10210 | 0.45 | 0.24 | 0 | 1 |
| Perceived social norm | 10210 | 0.49 | 0.2 | 0 | 1 |
| Trust in institutions | 10210 | 0.58 | 0.24 | 0 | 1 |
| Wave | 10210 | 4.61 | 2.24 | 1 | 8 |
| Wave: 1 (%) | 1025 | 10.04 |  |  |  |
| Wave: 2 (%) | 1288 | 12.62 |  |  |  |
| Wave: 3 (%) | 1277 | 12.51 |  |  |  |
| Wave: 4 (%) | 1296 | 12.69 |  |  |  |
| Wave: 5 (%) | 1379 | 13.51 |  |  |  |
| Wave: 6 (%) | 1326 | 12.99 |  |  |  |
| Wave: 7 (%) | 1336 | 13.09 |  |  |  |
| Wave: 8 (%) | 1283 | 12.57 |  |  |  |
| Gender | 10171 | 0.5 | 0.5 | 0 | 1 |
| Gender: male (%) | 5134 | 50.48 |  |  |  |
| Gender: female (%) | 5037 | 49.52 |  |  |  |
| Age | 10210 | 47.44 | 16.65 | 15 | 85 |
| Education | 10039 | 1.28 | 0.93 | 0 | 3 |
| Education: primary (%) | 1980 | 19.72 |  |  |  |
| Education: vocational (Lehre) (%) | 4700 | 46.82 |  |  |  |
| Education: secondary (%) | 1972 | 19.64 |  |  |  |
| Education: tertiary (%) | 1387 | 13.82 |  |  |  |
| Household size | 10115 | 1.5 | 1.24 | 0 | 5 |
| Migration background | 10045 | 0.18 | 0.39 | 0 | 1 |
| Migration background: no (%) | 8226 | 81.89 |  |  |  |
| Migration background: yes (%) | 1819 | 18.11 |  |  |  |
| Employment | 10210 | 2.1 | 1.74 | 0 | 4 |
| Employment: (self) employed as usual (%) | 3507 | 34.35 |  |  |  |
| Employment: short time work (%) | 615 | 6.02 |  |  |  |
| Employment: home office (%) | 1364 | 13.36 |  |  |  |
| Employment: unemployed (%) | 792 | 7.76 |  |  |  |
| Employment: not in labour market (%) | 3932 | 38.51 |  |  |  |
| Effectiveness of measures | 10210 | 0.56 | 0.29 | 0 | 1 |
| log(Regional 7day-incidence) | 10020 | 3.23 | 1.82 | 0 | 6.19 |
| Staying home | 10210 | 0.62 | 0.34 | 0 | 1 |
| Wearing mask | 10210 | 0.7 | 0.34 | 0 | 1 |
| Keeping distance | 10210 | 0.83 | 0.24 | 0 | 1 |
| 2019 general election vote | 8486 | 2.06 | 2.02 | 0 | 6 |
| 2019 general election vote: ÖVP (%) | 2597 | 30.6 |  |  |  |
| 2019 general election vote: SPÖ (%) | 1561 | 18.4 |  |  |  |
| 2019 general election vote: FPÖ (%) | 1303 | 15.35 |  |  |  |
| 2019 general election vote: GRÜNE (%) | 1139 | 13.42 |  |  |  |
| 2019 general election vote: NEOS (%) | 551 | 6.49 |  |  |  |
| 2019 general election vote: other (%) | 294 | 3.46 |  |  |  |
| 2019 general election vote: no vote (%) | 1041 | 12.27 |  |  |  |

Table AT1: Summary statistics of all variables used in the analyses.

Note: Refer to Appendix C1 for a detailed description of the indices of preventive behaviour, perceived health risks, perceived social norm, and trust in institutions. Refer to Appendix C2 for the full question wordings.

### A2. Full regression estimates

Besides providing estimates for the control variables, Table AT2, also displays the regression estimates of both the fractional model (6) and the tobit model (7). In sum, estimates indicate that our results are robust against alternative specifications of the link function and against alternative assumptions about the data structure. In fact, these approaches would suggest that the main effects of all variables of interest (perceived health risks, perceived social norm, and trust in institutions) and their interactions are even larger compared to our statistically more conservative approach of using 2FE (model 4).

|  | (1) | (2) | (3) | (4) | (5) | (6) | (7) |
| --- | --- | --- | --- | --- | --- | --- | --- |
|  | wave FE | wave FE | wave FE | 2FE | 2FE | FM | Tobit |
|  |  |  |  |  |  |  |  |
| Perceived health risks | 0.269^***^ | 0.689^***^ | 0.670^***^ | 0.385^***^ | 0.382^***^ | 1.040^***^ | 0.696^***^ |
|  | (0.0244) | (0.0425) | (0.0488) | (0.0651) | (0.0637) | (0.0911) | (0.0550) |
| Perceived social norm | 0.434^***^ | 0.763^***^ | 0.767^***^ | 0.608^***^ | 0.610^***^ | 1.704^***^ | 0.803^***^ |
|  | (0.0166) | (0.0437) | (0.0406) | (0.0364) | (0.0332) | (0.0774) | (0.0434) |
| Trust in institutions | 0.121^***^ | 0.173^**^ | 0.178^**^ | 0.107^+^ | 0.106^+^ | 0.443^***^ | 0.196^***^ |
|  | 0.269^***^ | 0.689^***^ | 0.670^***^ | 0.385^***^ | 0.382^***^ | 1.040^***^ | 0.696^***^ |
| Perceived social norm X Perceived health risks |  | -0.721^***^ | -0.709^***^ | -0.464^***^ | -0.468^***^ | -0.920^***^ | -0.640^***^ |
|  |  | (0.0769) | (0.0714) | (0.0535) | (0.0436) | (0.139) | (0.0761) |
| Trust in institutions X Perceived health risks |  | -0.147^*^ | -0.155^*^ | -0.119 | -0.112 | -0.300^*^ | -0.174^*^ |
|  |  | (0.0565) | (0.0648) | (0.0800) | (0.0772) | (0.121) | (0.0693) |
| log(Regional 7day-incidence) |  |  |  |  | 0.00339 |  |  |
|  |  |  |  |  | (0.00254) |  |  |
| Employment: short time work (ref.: employed) | -0.0175 | -0.0164 | -0.0110 | 0.00395 | 0.00323 | -0.0182 | -0.0132 |
|  | (0.0159) | (0.0147) | (0.0149) | (0.0104) | (0.00960) | (0.0236) | (0.0122) |
| Employment: home office | 0.0269^*^ | 0.0250^*^ | 0.0222^+^ | 0.0268^**^ | 0.0268^**^ | 0.0638^**^ | 0.0322^**^ |
|  | (0.00953) | (0.00879) | (0.00951) | (0.00691) | (0.00754) | (0.0211) | (0.0109) |
| Employment: unemployed | 0.0129 | 0.0131 | 0.00725 | 0.0175^*^ | 0.0184^*^ | 0.0346 | 0.0230 |
|  | (0.0159) | (0.0161) | (0.0164) | (0.00709) | (0.00597) | (0.0291) | (0.0160) |
| Employment: not in labour market | 0.0451^***^ | 0.0452^**^ | 0.0128 | 0.00658 | 0.000833 | 0.0438^+^ | 0.0199^*^ |
|  | (0.00834) | (0.00838) | (0.00840) | (0.0143) | (0.0145) | (0.0231) | (0.00972) |
| Measures are effective | 0.0476^+^ | 0.0354 | 0.0259 | -0.00166 | -0.00128 | 0.0393 | 0.0408^**^ |
|  | (0.0223) | (0.0222) | (0.0176) | (0.00775) | (0.00862) | (0.0267) | (0.0157) |
| Gender: female |  |  | 0.0508^***^ |  |  | 0.219^***^ | 0.0650^***^ |
|  |  |  | (0.00837) |  |  | (0.0223) | (0.00847) |
| Age |  |  | 0.00206^***^ |  |  | 0.00724^***^ | 0.00235^***^ |
|  |  |  | (0.000349) |  |  | (0.000769) | (0.000294) |
| Education: vocational (Lehre) (ref.: primary) |  |  | -0.0102 |  |  | -0.0401 | -0.0145 |
|  |  |  | (0.0109) |  |  | (0.0319) | (0.0118) |
| Education: secondary |  |  | 0.00594 |  |  | -0.0105 | 0.00682 |
|  |  |  | (0.0122) |  |  | (0.0363) | (0.0138) |
| Education: tertiary |  |  | 0.00516 |  |  | -0.000184 | 0.00603 |
|  |  |  | (0.0158) |  |  | (0.0404) | (0.0156) |
| Household size |  |  | -0.000643 |  |  | -0.00250 | -0.00239 |
|  |  |  | (0.00357) |  |  | (0.00911) | (0.00360) |
| Migration background: yes |  |  | -0.0172 |  |  | -0.0304 | -0.0174 |
|  |  |  | (0.0106) |  |  | (0.0289) | (0.0112) |
| 2019 general election vote: SPÖ (ref.: ÖVP) |  |  | -0.0118 |  |  |  |  |
|  |  |  | (0.0115) |  |  |  |  |
| 2019 general election vote: FPÖ |  |  | -0.0192 |  |  |  |  |
|  |  |  | (0.0129) |  |  |  |  |
| 2019 general election vote: Greens |  |  | 0.0265^+^ |  |  |  |  |
|  |  |  | (0.0125) |  |  |  |  |
| 2019 general election vote: NEOS |  |  | -0.00605 |  |  |  |  |
|  |  |  | (0.0146) |  |  |  |  |
| 2019 general election vote: other |  |  | 0.0153 |  |  |  |  |
|  |  |  | (0.0210) |  |  |  |  |
| 2019 general election vote: no vote |  |  | 0.0301^+^ |  |  |  |  |
|  |  |  | (0.0138) |  |  |  |  |
| Constant | 0.265^***^ | 0.0981^**^ | 0.00103 | 0.316^***^ | 0.308^***^ | -0.986^***^ | -0.0261 |
|  | (0.0201) | (0.0206) | (0.0332) | (0.0400) | (0.0424) | (0.0755) | (0.0361) |
| Observations | 10210 | 10210 | 8420 | 10210 | 10020 | 9976 | 9976 |

Table AT2: Full regression estimates: wave Fixed Effects (1, 2, 3), two-way Fixed Effects (4, 5), Fractional Model (6), and Tobit regression (7). Model (4) is the main model. Generally, models (1, 2, 4, 5) are equivalent to models (1, 2, 3, 4) in Table 1. Individual clustered standard errors in parenthesis (^+^ *p* < .10; ^*^ *p* < .05; ^**^ *p* < .01; ^***^ *p* < .001).

### A3. Single regressions: aspects of preventive behaviour (staying home, wearing masks, keeping distance)

The following table (AT3) provides separate wave fixed effects and two-way fixed effects regression estimates for each of the variables underlying the preventive behaviour index (*staying home*, *wearing mask*, and *keeping distance*). Each of these regressions on the single aspects of preventive behaviour includes sub-indices of the corresponding descriptive and injunctive norms and is estimated in the same way as the main regression models (Table 1, Models 2 and 3). Generally, the direct effects of the social norm variables and their interaction with perceived health risks remain quite robust in size and statistically significant. The interactions of trust in institutions and perceived health risks remain robust in size as well, albeit on a smaller level such that they are not always statistically significant.

|  | (1) | (2) | (3) | (4) | (5) | (6) |
| --- | --- | --- | --- | --- | --- | --- |
|  | wave FE  *staying home* | 2FE  *staying home* | wave FE  *wearing mask* | 2FE  *wearing mask* | wave FE  *keeping distance* | 2FE  *keeping distance* |
|  |  |  |  |  |  |  |
| Perceived health risks | 0.648^***^ | 0.348^**^ | 0.641^***^ | 0.363^***^ | 0.682^***^ | 0.401^***^ |
|  | (0.0407) | (0.0956) | (0.0437) | (0.0464) | (0.0629) | (0.0727) |
| Trust in institutions | 0.215^***^ | 0.147^+^ | 0.128^*^ | 0.129^+^ | 0.198^***^ | 0.0776^+^ |
|  | (0.0380) | (0.0751) | (0.0493) | (0.0589) | (0.0347) | (0.0345) |
| Trust in institutions X | -0.201^*^ | -0.130 | -0.121 | -0.141 | -0.191^**^ | -0.148^*^ |
| Perceived health risks | (0.0665) | (0.121) | (0.0799) | (0.0886) | (0.0543) | (0.0573) |
| Norms: staying home | 0.700^***^ | 0.576^***^ |  |  |  |  |
|  | (0.0323) | (0.0392) |  |  |  |  |
| Norms: staying home X | -0.603^***^ | -0.436^**^ |  |  |  |  |
| Perceived health risks | (0.0744) | (0.0838) |  |  |  |  |
| Norms: wearing mask |  |  | 0.959^***^ | 0.689^***^ |  |  |
|  |  |  | (0.0405) | (0.0356) |  |  |
| Norms: wearing mask X |  |  | -0.595^***^ | -0.412^***^ |  |  |
| Perceived health risks |  |  | (0.0670) | (0.0533) |  |  |
| Norms: keeping distance |  |  |  |  | 0.613^***^ | 0.398^***^ |
|  |  |  |  |  | (0.0419) | (0.0291) |
| Norms: keeping distance X |  |  |  |  | -0.726^***^ | -0.391^***^ |
| Perceived health risks |  |  |  |  | (0.0726) | (0.0559) |
| Employment: short time | -0.00858 | 0.0203 | -0.0245 | -0.00647 | -0.0209 | -0.00161 |
| work (Ref. Employed) | (0.0198) | (0.0131) | (0.0204) | (0.0150) | (0.0145) | (0.0125) |
| Employment: home office | 0.0639^**^ | 0.0668^***^ | -0.00180 | -0.00706 | 0.0125 | 0.0218 |
|  | (0.0129) | (0.0110) | (0.0128) | (0.0109) | (0.00926) | (0.0131) |
| Employment: unemployed | 0.0374^+^ | 0.0128 | 0.000243 | 0.0166 | -0.00518 | 0.0286^*^ |
|  | (0.0184) | (0.0117) | (0.0192) | (0.0134) | (0.0159) | (0.00966) |
| Employment: not in labour | 0.0676^***^ | 0.0306 | 0.0297^*^ | -0.00493 | 0.0322^**^ | -0.00232 |
| market | (0.0113) | (0.0182) | (0.0105) | (0.0175) | (0.00870) | (0.0159) |
| Perceived effectiveness of | 0.0290 | -0.0183 | 0.0396 | 0.0110 | 0.0363 | 0.0102 |
| measures | (0.0254) | (0.0146) | (0.0212) | (0.0138) | (0.0264) | (0.00774) |
| Constant | 0.0564^*^ | 0.268^**^ | -0.0284 | 0.229^***^ | 0.269^***^ | 0.512^***^ |
|  | (0.0235) | (0.0544) | (0.0215) | (0.0409) | (0.0304) | (0.0413) |
| Observations | 10210 | 10210 | 10210 | 10210 | 10210 | 10210 |

Table AT3: Results of different regression models (wave Fixed Effects and two-way Fixed Effects) focusing on the single behavioural variables underlying the preventive behaviour index: staying home (1, 2), wearing mask (3, 4), and keeping distance (5, 6). Each regression includes sub-indices of descriptive and injunctive norms (Norms: staying home; Norms: wearing mask; Norms: keeping distance) corresponding to each dependent variable. Reference category for employment status is standard employment. Standard errors in parenthesis (^+^ *p* < .10; ^*^ *p* < .05; ^**^ *p* < .01; ^***^ *p* < .001).

## S1 Appendix B. Checks of model assumptions and robustness of the moderation

### B1. Test linearity of moderation effects

We apply a binning estimator to check whether the multiplicative interactions of risk perceptions, on the one hand, and trust in institutions or social norms, on the other hand, are linear. The analyses were carried out by the STATA command *interflex* (see Hainmueller et al., 2019). As suggested by Hainmueller et al., we use three bins at a low, medium, and high level of the moderator (median values of tertiles of perceived health risks). As Fig BF1 shows, the binning estimator of all points of the interaction between perceived health risks and perceived social norms remain quite close to the prediction of a linear interaction. Contrary to that, the binning estimator of the interaction between perceived health risks and trust in institutions indicates possible non-linearities. Wald tests provide p-values of .33 (social norms) and .04 (trust in institutions), showing that the NULL-hypothesis (that the binning estimator is statistically equivalent to the linear interaction model) is rejected for the interaction of trust in institutions and risk perceptions.

To further investigate the functional form of the moderations, we calculate the interactions using a kernel estimator (Figs BF3 and BF4). Again, we find that the interaction between social norms and perceived health risks remains linear for the majority of the distribution of the moderator. The size of these moderations is lower than a linear model would predict only for observations that feature very high and very low risk perceptions. BF4 showing the interaction between perceived risks and trust in institutions suggests a more non-linear relationship. Here we see that trust affects participants’ average preventive behaviour positively only at medium levels of perceived health risks. However, in contrast to the predictions of a linear model, using a kernel estimator suggests that this effect of trust remains close to zero when perceived risk levels are higher or lower. In sum these results suggest that the interaction between social norms and risk perceptions is approximately linear, while we find more variation in the size of the interaction between trust in institutions and risk perceptions. Kernel estimators suggest that trust in institutions fail to facilitate preventive behaviour when levels of perceived health risks are low, while social norms remain important facilitators of preventive behaviour. However, further experimental research is necessary to test this non-linear relationship because our sample has only a limited number of respondents with very low and high levels of trust in institutions.

Binning estimator


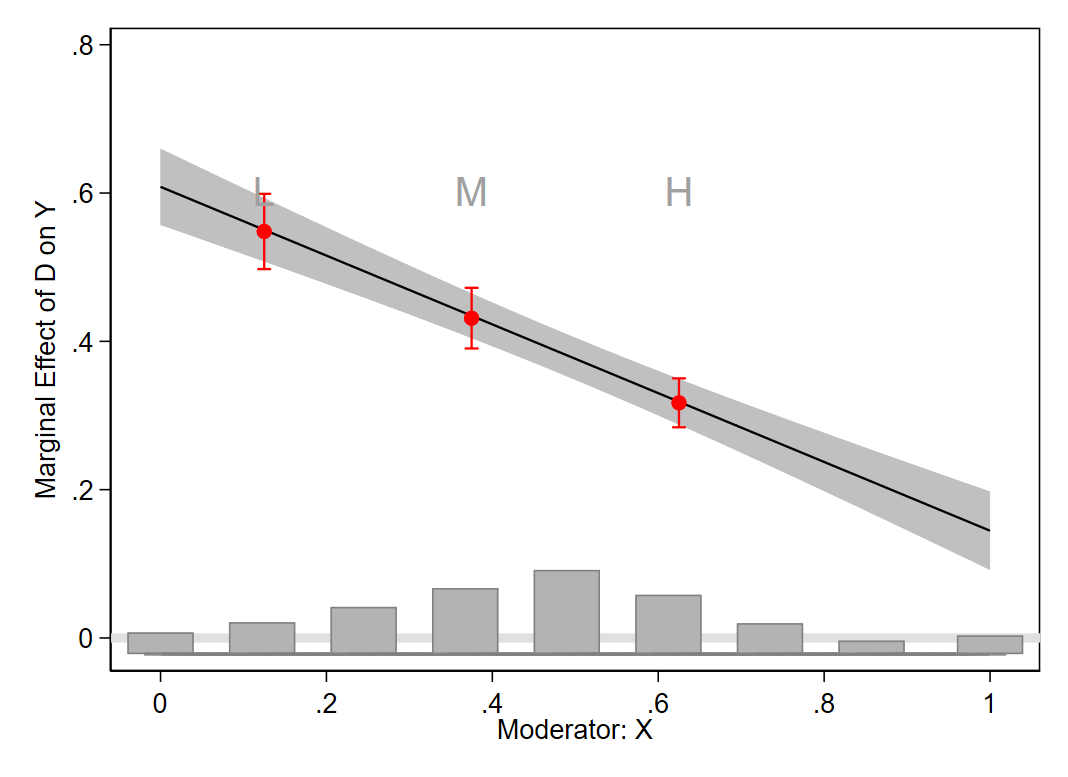


Fig BF1: Marginal effect of social norms (D) on preventive behaviour (Y), moderated by perceived health risks (X). Point estimates are based on a binning estimator for important values in the distribution (the median values of the tertiles of the moderator variable). The histogram shows the distribution of perceived health risks in our sample. The regression includes all variables of model 3 in Table 1 in the main text. Linear Fixed Effects model with wave as well as individual fixed effects. 95% confidence intervals (grey areas) are calculated using individually clustered standard errors.


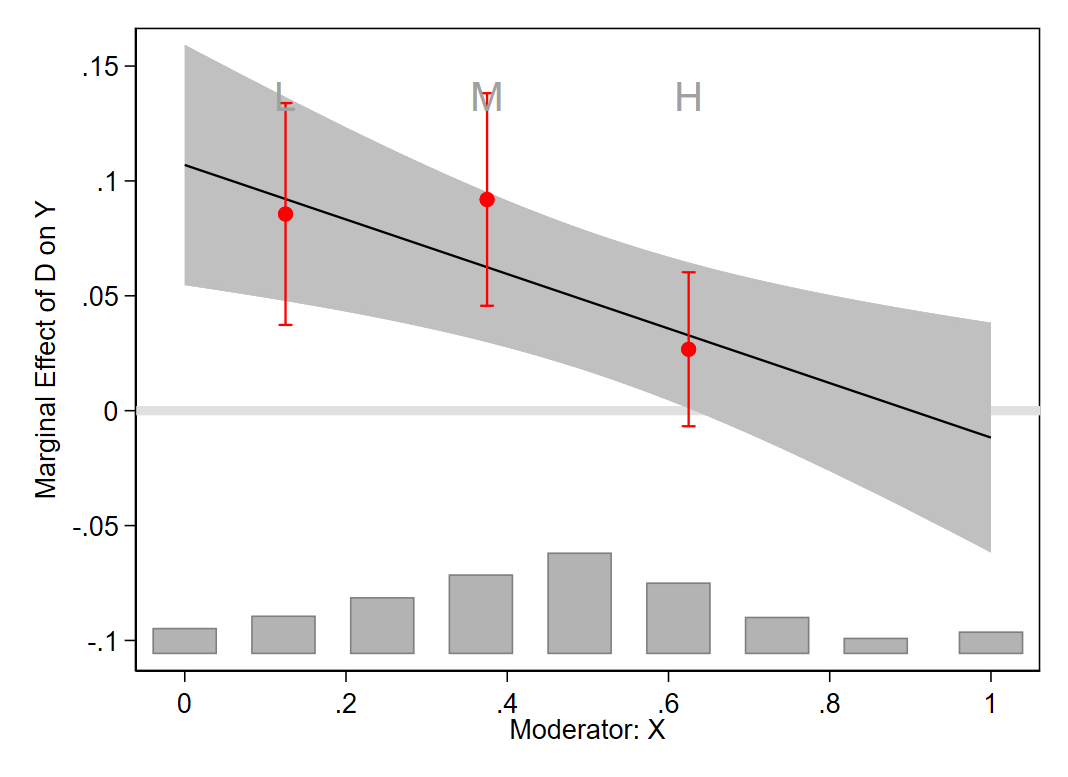


Fig BF2: Marginal effect of trust in institutions (D) on preventive behaviour (Y), moderated by perceived health risks (X). Point estimates are based on a binning estimator for important values in the distribution (the median values of the tertiles of the moderator variable). The histogram shows the distribution of perceived health risks in our sample. The regression includes all variables of model 3 in Table 1 in the main text. Linear Fixed Effects model with wave as well as individual fixed effects. 95% confidence intervals (grey areas) are calculated using individually clustered standard errors.

Kernel estimator


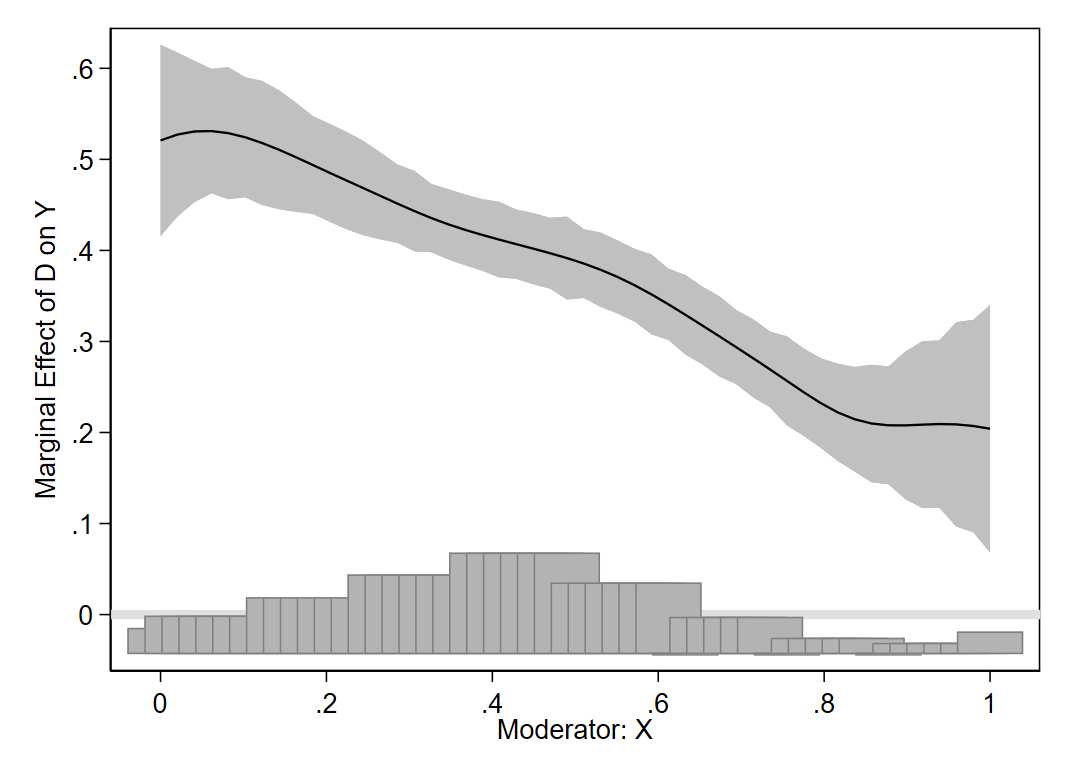


Fig BF3 Marginal effect of social norms on preventive behaviour, moderated by perceived health risks. Estimates show results of a kernel estimator. The histogram shows the distribution of perceived health risks in our sample. The regression includes all variables of model 3 in Table 1 in the main text. Linear Fixed Effects model with wave as well as individual fixed effects. 95% confidence intervals (grey areas) are calculated using bootstrapped standard errors.


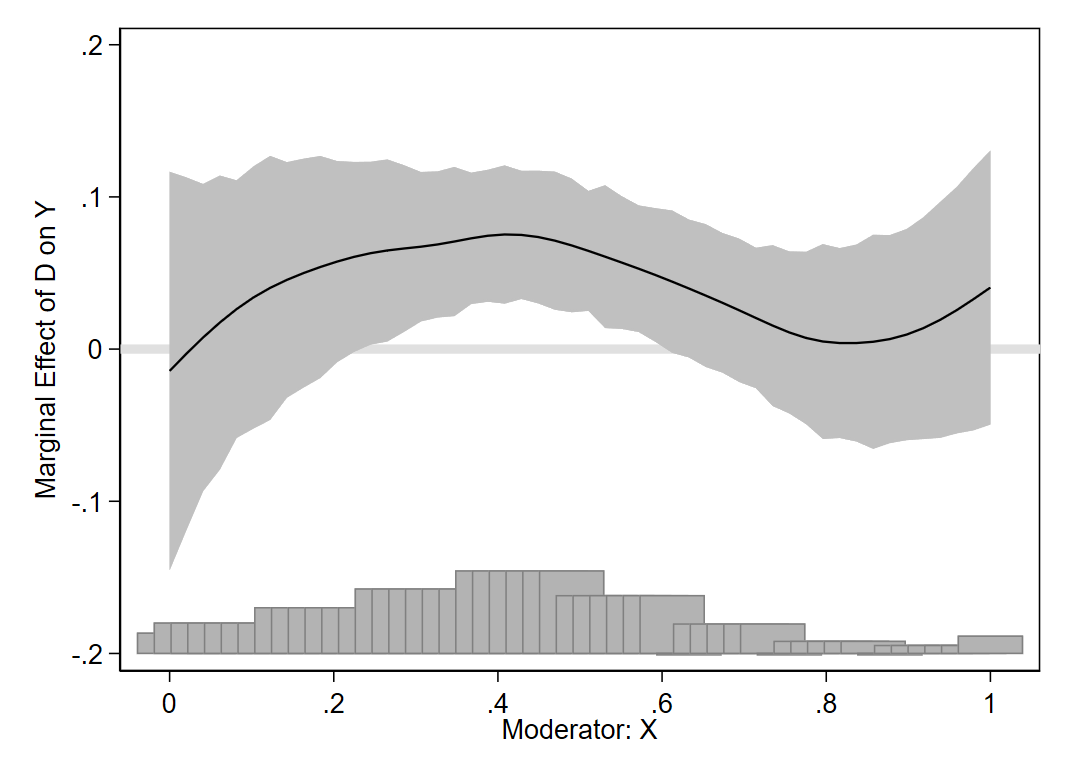


Fig BF4: Marginal effect of trust in institutions on preventive behaviour, moderated by perceived health risks. Estimates show results of a kernel estimator. The histogram shows the distribution of perceived health risks in our sample. The regression includes all variables of model 3 in Table 1 in the main text. Linear Fixed Effects model with wave as well as individual fixed effects. 95% confidence intervals (grey areas) are calculated using bootstrapped standard errors.

### B2. Check robustness of moderation against sample restrictions

To show the robustness of our estimates regarding sample restrictions, we rerun the full model while excluding all observations one wave at a time. This also tests whether our results depend on one outlying wave, which would suggest that the observed relationships are not stable over time but rather depend on specific circumstances. Figs BF5 and BF6 demonstrate that the size and direction of the moderation of social norms as well as trust in institutions by perceived health risks is not substantially affected by this exercise. Focusing again on the relative change in the size of the effects of social norms and trust in institutions from moderately low to moderately high levels of perceived risks (one within standard deviation above and below the mean), we find that increasing levels of risk reduce the effect of social norms by 23% – 27% (full sample: 25%) and reduce the effect of trust in institutions by 25% – 50% (full sample: 43%). The latter effect, however, is much smaller in size. In sum, excluding specific waves from our analysis changes little in the interaction between social norms and perceived health risks, suggesting that the findings are robust to sample restrictions and do not depend on single wave outliers.


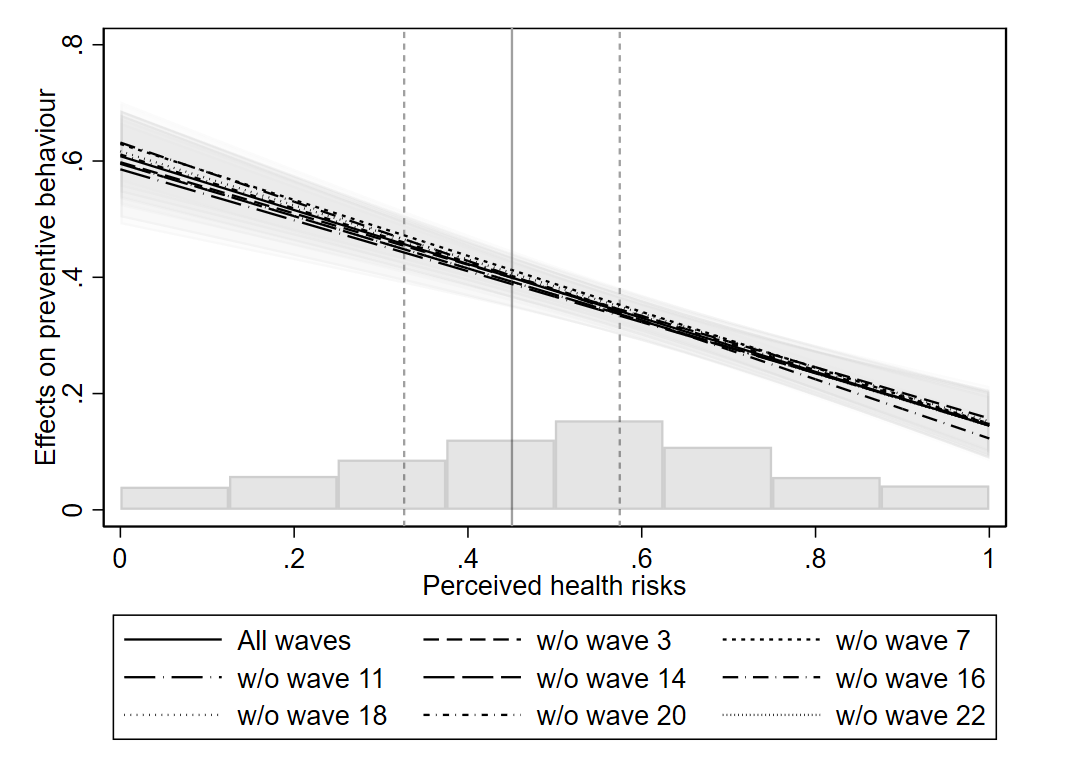


Fig BF5: Average marginal effects of social norms on preventive behaviour depending on level of risk by sample. Predictions based on estimates in model 3 in Table 1 in the main text (all waves) and similar models depending on sample restrictions as specified in the legend. Linear Fixed Effects model with wave as well as individual fixed effects. 95% confidence intervals (grey areas) are calculated using two-way clustered standard errors for individuals and waves. The histogram represents the distribution of social norm perceptions in the full sample. Dashed lines mark +/- one within-individual SD from the mean.


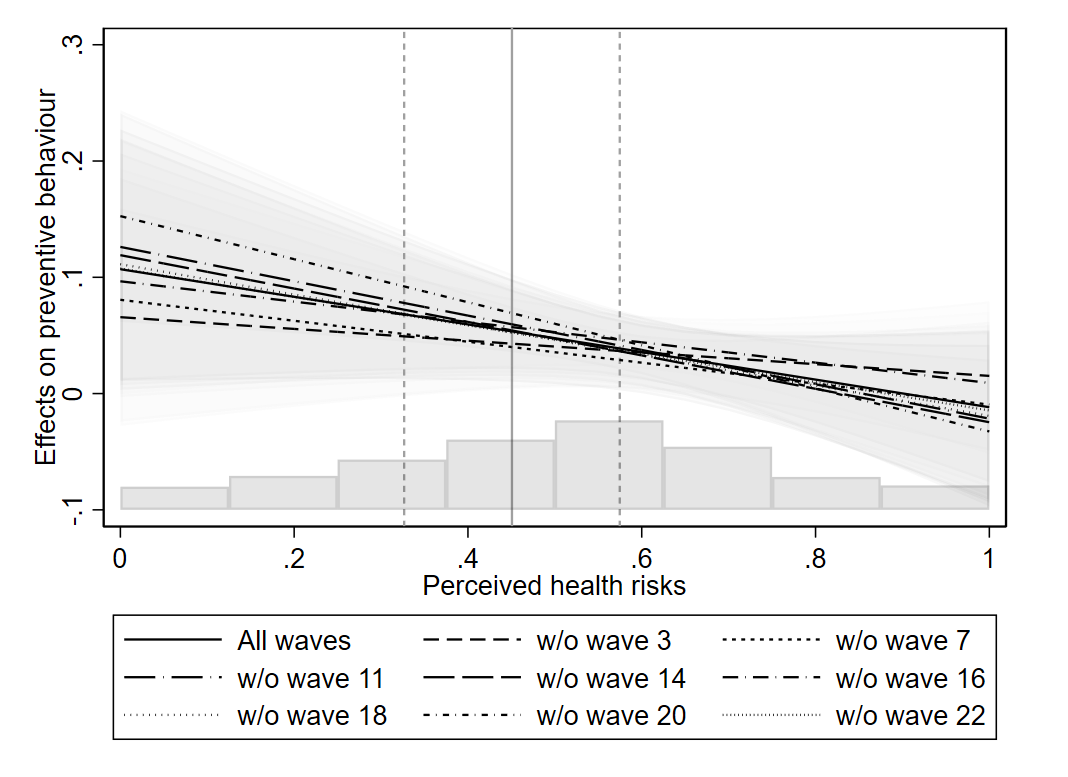


Fig BF6: Average marginal effects of trust in institutions on preventive behaviour depending on level of risk by sample. Predictions based on estimates in model 3 in Table 1 in the main text (all waves) and similar models depending on sample restrictions as specified in the legend. Linear Fixed Effects model with wave as well as individual fixed effects. 95% confidence intervals (grey areas) are calculated using two-way clustered standard errors for individuals and waves. The histogram represents the distribution of social norm perceptions in the full sample. Dashed lines mark +/- one within-individual SD from the mean.

### B3. Check of parallel trends assumption

The following table (BT1) provides the information for assessing the plausibility of the parallel trends assumption, immanent in analyses using two way fixed effects estimators (Angrist and Pischke, 2008). First, we add a lead on the effect of the three independent variables of perceived health risks, social norms, and trust in institutions (model 2) and compare it to our main results (model 1). All three leads are insignificant and marginal in size, which is in line with the parallel trends assumption. Second, we include individual-specific time trends (model 3). As Table BT1 shows, the sizes of the social norms coefficients decrease slightly compared to our main model (1) but remain substantial in size. This again provides evidence that the parallel trends assumption is plausible for this effect. The estimates of the main effect of trust in institutions and its interaction with perceived health risks lose statistical significance in model 3. This suggests that it is unclear whether the parallel trends assumption holds in the case of trust in institutions, and again emphasizes the smaller effect size of trust compared to social norms.

|  | (1) | (2) | (3) |
| --- | --- | --- | --- |
|  | 2FE | 2FE check 1 | 2FE check 2 |
|  |  |  |  |
| Perceived health risks | 0.385^***^ | 0.360^***^ | 0.285^***^ |
|  | (0.0651) | (0.0395) | (0.0549) |
|  |  |  |  |
| Perceived social norm | 0.608^***^ | 0.590^***^ | 0.563^***^ |
|  | (0.0364) | (0.0304) | (0.0399) |
|  |  |  |  |
| Trust in institutions | 0.107^+^ | 0.102^**^ | 0.0573 |
|  | (0.0509) | (0.0320) | (0.0451) |
|  |  |  |  |
| Perceived social norm X Perceived health risks | -0.464^***^ | -0.431^***^ | -0.377^***^ |
|  | (0.0535) | (0.0532) | (0.0726) |
|  |  |  |  |
| Trust in institutions X Perceived health risks | -0.119 | -0.106^*^ | -0.0457 |
|  | (0.0800) | (0.0498) | (0.0718) |
|  |  |  |  |
| Lead: Perceived health risks |  | 0.00497 | 0.00249 |
|  |  | (0.0150) | (0.0212) |
|  |  |  |  |
| Lead: Perceived social norm |  | -0.000500 | -0.00959 |
|  |  | (0.0146) | (0.0218) |
|  |  |  |  |
| Lead: Trust in institutions |  | -0.00439 | -0.000612 |
|  |  | (0.0189) | (0.0299) |
|  |  |  |  |
| Controls | Yes | Yes | Yes |
| Wave X id | No | No | Yes |
| Observations | 10210 | 6679 | 6679 |

Table BT1: Parallel trends assumption checks. Model (1) represents our main model similar to Model 3 Table 1 in the main manuscript, model (2) includes leads of our three independent variables, and model (3) additionally includes individual-specific time trends. Controls include dummies for employment status and a variable indicating perceived effectiveness of governmental measures (see Methods). Standard errors clustered by id in parenthesis (^+^ *p* < .10; ^*^ *p* < .05; ^**^ *p* < .01; ^***^ *p* < .001).

### B4. Balance checks of data

The tests were conducted in STATA by the *ietoolkit*

(<https://blogs.worldbank.org/impactevaluations/ie-analytics-introducing-ietoolkit>).

See the STATA code provided online for further details in Appendix D2.

|  |  | (1) |  | (2) | t-test |
| --- | --- | --- | --- | --- | --- |
|  |  | *included* |  | *excluded* | Difference |
| Variable | N/[Clusters] | Mean/[SE] | N/[Clusters] | Mean/[SE] | (1)-(2) |
| Preventive behaviour | 9811 | 0.715 | 2124 | 0.725 | -0.010 |
|  | [1914] | [0.005] | [1173] | [0.008] |  |
| Trust in institutions | 9811 | 0.586 | 1695 | 0.493 | 0.093*** |
|  | [1914] | [0.005] | [931] | [0.009] |  |
| Perceived social norms | 9811 | 0.488 | 1766 | 0.509 | -0.021* |
|  | [1914] | [0.003] | [1043] | [0.006] |  |
| Perceived health risks | 9811 | 0.450 | 2480 | 0.442 | 0.008** |
|  | [1914] | [0.005] | [1246] | [0.007] |  |
| Gender | 9771 | 0.495 | 2590 | 0.549 | -0.054*** |
|  | [1906] | [0.012] | [1260] | [0.017] |  |
| Age | 9811 | 47.827 | 2600 | 39.820 | 8.007*** |
|  | [1914] | [0.402] | [1264] | [0.587] |  |
| Education | 9655 | 1.272 | 2499 | 1.185 | 0.087** |
|  | [1877] | [0.023] | [1225] | [0.033] |  |
| Household size | 9727 | 1.488 | 2568 | 1.704 | -0.216*** |
|  | [1891] | [0.030] | [1248] | [0.047] |  |
| Employment: (self) employed as usual | 9663 | 0.182 | 2483 | 0.209 | -0.027* |
|  | [1877] | [0.010] | [1219] | [0.015] |  |
| Employment: short time work | 9811 | 0.341 | 2475 | 0.337 | 0.004 |
|  | [1914] | [0.010] | [1237] | [0.015] |  |
| Employment: home office | 9811 | 0.061 | 2475 | 0.059 | 0.002 |
|  | [1914] | [0.004] | [1237] | [0.007] |  |
| Employment: unemployed | 9811 | 0.134 | 2475 | 0.109 | 0.025*** |
|  | [1914] | [0.007] | [1237] | [0.009] |  |
| Employment: not in labour market | 9811 | 0.078 | 2475 | 0.099 | -0.021** |
|  | [1914] | [0.005] | [1237] | [0.009] |  |
| Migration background | 9811 | 0.387 | 2475 | 0.396 | -0.010 |
|  | [1914] | [0.012] | [1237] | [0.017] |  |
| Perceived effectiveness of measures | 9811 | 0.566 | 2201 | 0.557 | 0.009** |
|  | [1914] | [0.005] | [1184] | [0.007] |  |

Table BT2 Balance checks between retained and dropped observations of the dependent, independent, and control variables. The value displayed for t-tests are the differences in the means across the groups. Standard errors are clustered at participant level. Fixed effects using survey waves are included in all estimated t-tests. Number of clusters and standard errors in parenthesis (^*^ *p* < 0.05, ^**^ *p* < 0.01, ^***^ *p* < 0.001).

### B5. Comparison of survey and mobile data (external validity)


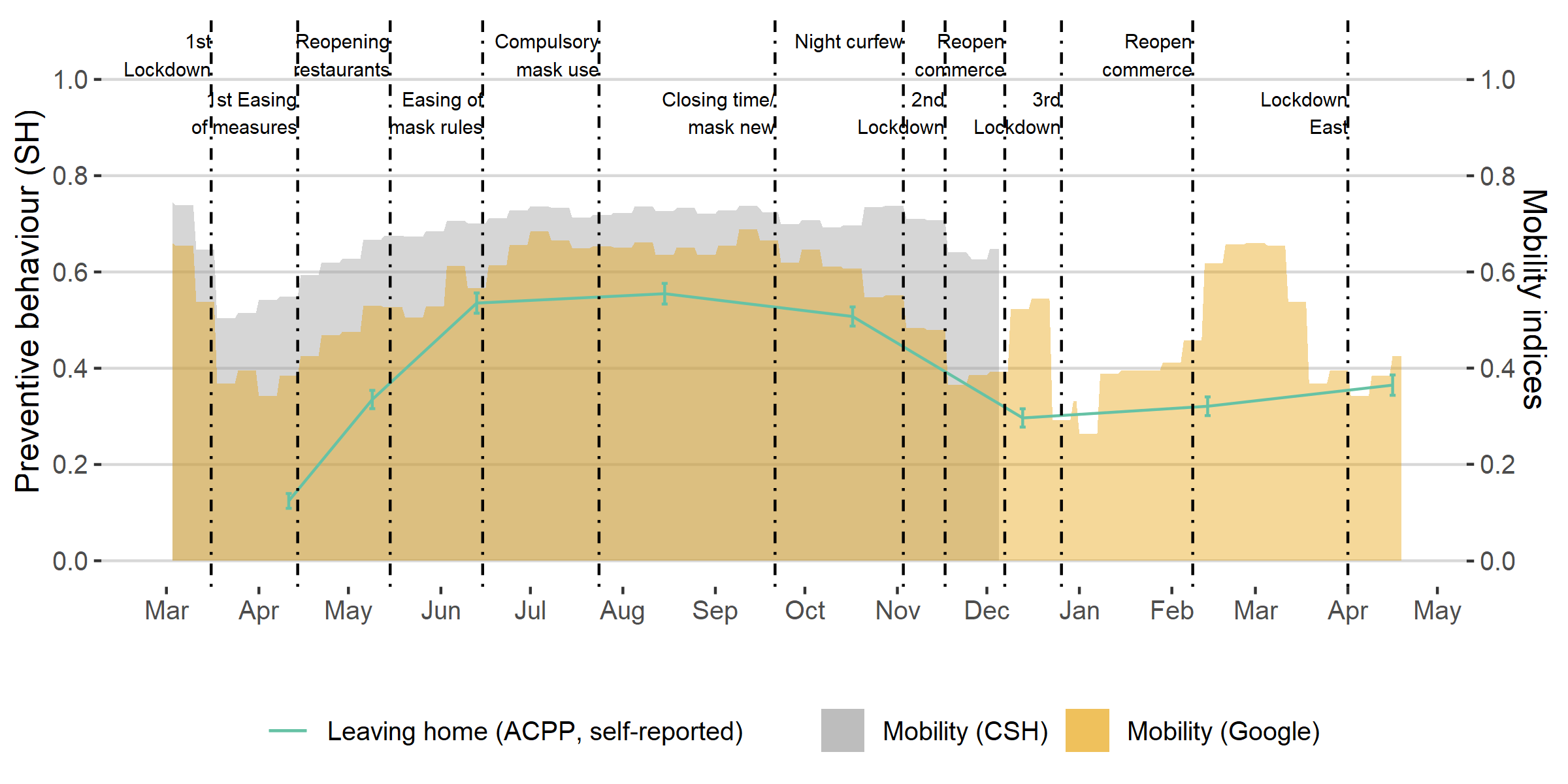


Fig BF7: Evolution of average self-reported propensity to leave one’s home (disagreement to the statement “stay at home except for necessities” measured by a 5-level Likert scale ranging from “almost never” to “almost always”), the Google mobility index, and mobility data from CSH (Complexity Science Hub Vienna) over the course of the pandemic. Note: “Staying at home” is one of three dimensions of the preventive behaviour index used in the main analyses (see Methods and Appendix C1). Google COVID-19 Community Mobility Reports are publicly available at (Google, 2020). Our google mobility measure is a summarised index of movement pattern changes in retail and recreation, grocery and pharmacy, transit stations, workplaces, and residential areas (reversed) per week. The numbers reflect relative changes compared to the baseline measure: the average movement in the first week of the Google dataset (week 6 of 2020). The CSH mobility averages are an updated version of Fig 4 Panel A in Heiler et al. (2020).^[[1]](#footnote-1)^ The indices indicate the share of mobile phone devices with a ROG (radius of gyration) between 0 and 500 meters in Austria per week. Thus, the CSH mobility measure approximates the share of people who did not leave their neighbourhood in Austria. Remaining dissimilarities between Google GPS data and ACPP survey data in December 2020 may result from differences in the measurement strategies: while the Google GPS data captures mobility at a certain date, our survey measures ask respondents to report how frequently they left the house in the last week. Thus, the mobility captured by the survey data should lag behind. This might explain the lower mobility reported in the survey in December and February shortly after commerce reopened.

### B6. Alternative operationalization of preventive behaviour and placebo check

To check whether or not our results depend on similarities between the measurement instruments of perceived social norms and preventive behaviour (refer to question wording in Appendix C2) and their close proximity in the questionnaire (which could lead to anchoring bias), we use an alternative variable to operationalize preventive behaviour. This variable measures the amount of nonessential mobility and consists of an additive index of respondents’ self-reported frequency of going out (i) to meet friends and (ii) because they were bored. Both behaviours were strongly discouraged by the government or, in the case of meeting friends, even banned for an extended period of time during the pandemic (with minor exceptions). We invert the variable to get a measure indicating how strongly people avoid nonessential mobility. Table BT3 shows estimates from a 2FE regression with similar specifications compared to our main model (Table 1 in the main text, model 4). The coefficients suggest that our conclusion also holds when using a different variable to indicate preventive behaviour as perceived health risk, social norms, and trust in institutions have positive effects on preventive behaviour, and the effects of perceived social norms and trust in institutions decrease when levels of perceived health risks increase.

In a second step, we constructed a variable indicating whether people avoided essential mobility and again tested whether our main variables of interest would affect this behaviour. This approach enables us to check if our variables of interest also affect behaviours that are not banned or discouraged. Hence, this regression essentially provides a placebo check for our results, and we do not expect to find any sizeable effects. The variable indicating whether people avoided essential mobility consists of an additive index of the inverted answers to questions about how often respondents went out (i) to buy groceries or (ii) to buy medicine or to visit a doctor. Model 2 shows the estimates of this regression. In line with our expectations, all the main effects are small and fail to reach statistically significant levels. The same is the case with the interaction effects. Although the interaction between social norms and health risks reaches weak levels of statistical significance (p<.1), the effect points in the opposite direction than its equivalent in our main model. Thus, as expected, the placebo check indicates that the effects we describe are limited to discouraged or banned behaviours during the pandemic.

|  | (1) | (2) |
| --- | --- | --- |
|  | 2FE Avoiding unessential mobility | 2FE Avoiding essential mobility |
| Perceived health risks | 0.221^***^ | -0.0107 |
|  | (0.0361) | (0.0323) |
| Perceived social norm | 0.115^**^ | 0.000436 |
|  | (0.0316) | (0.0193) |
| Trust in institutions | 0.0744^*^ | -0.00466 |
|  | (0.0288) | (0.0269) |
| Perceived social norm X | -0.138^*^ | 0.0680^+^ |
| Perceived health risks | (0.0518) | (0.0343) |
| Trust in institutions X | -0.0923^+^ | -0.00346 |
| Perceived health risks | (0.0479) | (0.0420) |
| Employment: short time work | -0.0113 | 0.00311 |
|  | (0.00971) | (0.00830) |
| Employment: home office | -0.00307 | 0.00480 |
|  | (0.00692) | (0.00621) |
| Employment: unemployed | -0.0355^*^ | 0.00473 |
|  | (0.0124) | (0.0113) |
| Employment: not in labour | -0.0148 | -0.000192 |
| market | (0.0134) | (0.00986) |
| Measures are effective | 0.00585 | 0.0104 |
|  | (0.0102) | (0.00648) |
| Constant | 0.626^***^ | 0.731^***^ |
|  | (0.0237) | (0.0158) |
| Observations | 10021 | 10076 |

Table BT3: Full regression estimates: OLS with two-way Fixed Effects. Individual clustered standard errors in parenthesis (+ p < .10; * p < .05; ** p < .01; *** p < .001).

### B7. Testing the influence of working from home

We include working from home here because we focused on the mostly “involuntary” aspect of this infection prevention strategy, which is in part a decision made by the employer. However, since many employees could to some extent decide on the frequency of working from home, we should not control for it. To check whether this biases our estimates we calculate the results of regression models excluding the dummy on working from home. Comparing the coefficient estimates reported in Table BT4 to those reported in Table 1 in the main manuscript, we find that excluding this dummy does not substantially change any of the main effects we are interested in.

|  | (1) | (2) | (3) | (4) |
| --- | --- | --- | --- | --- |
|  | Preventive behaviour | Preventive behaviour | Preventive behaviour | Preventive behaviour |
| Perceived health risks | 0.269^***^ | 0.696^***^ | 0.385^***^ | 0.383^***^ |
|  | (0.0245) | (0.0416) | (0.0653) | (0.0637) |
|  |  |  |  |  |
| Perceived social norm | 0.440^***^ | 0.770^***^ | 0.610^***^ | 0.612^***^ |
|  | (0.0170) | (0.0419) | (0.0360) | (0.0329) |
|  |  |  |  |  |
| Trust in institutions | 0.146^***^ | 0.196^***^ | 0.107^+^ | 0.106^+^ |
|  | (0.0197) | (0.0352) | (0.0518) | (0.0493) |
|  |  |  |  |  |
| Perceived social norm X Perceived health risks |  | -0.727^***^ | -0.466^***^ | -0.470^***^ |
|  |  | (0.0761) | (0.0535) | (0.0441) |
|  |  |  |  |  |
| Trust in institutions X Perceived health risks |  | -0.157^*^ | -0.117 | -0.110 |
|  |  | (0.0558) | (0.0796) | (0.0766) |
|  |  |  |  |  |
| log(Regional 7day-incidence) |  |  |  | 0.00348 |
|  |  |  |  | (0.00252) |
| Controls w/o Home office | Yes | Yes | Yes | Yes |
| Wave FE | Yes | Yes | Yes | Yes |
| Individual FE | No | No | Yes | Yes |
| Observations | 10210 | 10210 | 10210 | 10020 |
| Individuals | 2030 | 2030 | 2030 | 1983 |

Table BT4: Regression estimates: OLS with Fixed Effects. Individual clustered standard errors in parenthesis (+ p < .10; * p < .05; ** p < .01; *** p < .001).

### B8. Assessing multicollinearity between perceived social norms and perceived health risks and testing for potential mediating effects

To get a first impression of the potential size of multicollinearity due to social norms and risk perceptions we calculated the Pearson correlation coefficient within each wave. These coefficients indicate that the correlation varies between .14 and .25. This correlation is also visible when we regress social norms on risk perceptions. With rising levels of social norms, risk levels rise on average by factor of .26 (Table BT5, model 1). However, we argue that this relationship is mainly driven by time-invariant confounders. If we account for this issue by including individual fixed effects, the coefficient decreases to .07 (Table BT5, model 2). This correlation is still statistically significant but its size is not substantial anymore. Thus, our strategy of using 2-FE substantially reduces the potential for biases due to multicollinearity.

In addition, we test whether the exclusion of perceived risks in our main models would substantially decrease the effect of social norms on preventive behaviour, which would be the expected outcome if social norms negatively affect perceived health risks. This is not the case as Table BT5, model 3-6 shows. Neither the comparison between models 3 and 4, which only include wave FE, nor the comparison between models 5 and 6, which include both FE, indicate that the inclusion of perceived health risks leads to an overestimation of the effect of perceived social norms on preventive behaviour. As expected the coefficients change even less in the 2-FE model due to the reduced size of correlation between perceived social norms and perceived health risks.

|  | (1) | (2) | (3) | (4) | (5) | (6) |
| --- | --- | --- | --- | --- | --- | --- |
|  | Perceived health risks | Perceived health risks | Preventive behaviour | Preventive behaviour | Preventive behaviour | Preventive behaviour |
| Perceived social norm | 0.263^***^ | 0.0749^**^ | 0.485^***^ | 0.434^***^ | 0.406^***^ | 0.400^***^ |
|  | (0.0242) | (0.0165) | (0.0200) | (0.0166) | (0.0221) | (0.0211) |
|  |  |  |  |  |  |  |
| Trust in institutions |  |  | 0.177^***^ | 0.121^***^ | 0.0590^*^ | 0.0543^*^ |
|  |  |  | (0.0209) | (0.0173) | (0.0203) | (0.0198) |
|  |  |  |  |  |  |  |
| Employment: short time work |  |  | -0.00654 | -0.0175 | 0.00578 | 0.00415 |
|  |  |  | (0.0153) | (0.0159) | (0.0112) | (0.0107) |
|  |  |  |  |  |  |  |
| Employment: home office |  |  | 0.0293^*^ | 0.0269^*^ | 0.0286^**^ | 0.0276^**^ |
|  |  |  | (0.0106) | (0.00953) | (0.00714) | (0.00704) |
|  |  |  |  |  |  |  |
| Employment: unemployed |  |  | 0.0185 | 0.0129 | 0.0190^*^ | 0.0201^*^ |
|  |  |  | (0.0151) | (0.0159) | (0.00692) | (0.00705) |
|  |  |  |  |  |  |  |
| Employment: not in labour market |  |  | 0.0525^***^ | 0.0451^***^ | 0.00651 | 0.00455 |
|  |  |  | (0.00887) | (0.00834) | (0.0149) | (0.0143) |
|  |  |  |  |  |  |  |
| Measures are effective |  |  | 0.0425^+^ | 0.0476^+^ | -0.00301 | -0.000399 |
|  |  |  | (0.0223) | (0.0223) | (0.00799) | (0.00799) |
|  |  |  |  |  |  |  |
| Perceived health risks |  |  |  | 0.269^***^ |  | 0.0867^**^ |
|  |  |  |  | (0.0244) |  | (0.0169) |
| Wave FE | Yes | Yes | Yes | Yes | Yes | Yes |
| Individual FE | No | Yes | No | No | Yes | Yes |
| Observations | 10210 | 10210 | 10210 | 10210 | 10210 | 10210 |
| Individuals | 2030 | 2030 | 2030 | 2030 | 2030 | 2030 |

Table BT5: Regression estimates: OLS with Fixed Effects. Individual clustered standard errors in parenthesis (^+^ *p* < .10; ^*^ *p* < .05; ^**^ *p* < .01; ^***^ *p* < .001).

## S1 Appendix C. Variables, indices, and question wordings

### C1. Variables and indices

To operationalize the core variables, we use additive indices. For better comparison, these indices have been normalized so that each ranges from 0 – 1. We also conducted principal components analyses (PCAs) for every set of variables underlying the indices, and calculated correlation coefficients between the first components and the additive indices to check validity. The results are discussed in the following.

Preventive behaviour (DV)

A PCA shows that one component is sufficient to condense the three underlying variables *staying at home except for necessities*, *keeping distance of at least 1 meter*, and *wearing masks whenever distance cannot be maintained* (see Fig CF1). The first component loads all three variables fairly equally (see Table CT1), and the correlation coefficient with the additive index is 0.92.

|  | Comp1 | Comp2 | Comp3 |
| --- | --- | --- | --- |
| Wearing mask | 0.574 | -0.670 | 0.472 |
| Staying home | 0.570 | 0.740 | 0.357 |
| Keeping distance | 0.588 | -0.064 | -0.806 |

Table CT1: Principal components of the preventive behaviour index. The first component is considered sufficient, as the scree plot of eigenvalues in Fig CF1 shows.

Fig CF1: Scree plot of eigenvalues after principal component analysis for the preventive behaviour index.

Trust in institutions (IV)

According to the PCA, one component is sufficient for aggregating the four variables *trust in the government*, *trust in the health care system*, *trust in the parliament*, and *trust in the police* (see Fig CF2). The first component loads fairly equally on all underlying variables (see Table CT2) and the correlation coefficient with the additive index is 0.92. It is worth noting that the items on trust in public institutions have not been asked in wave three. To provide a continuous picture, we imputed the values for this wave as the means of waves two and four.

|  | Comp1 | Comp2 | Comp3 | Comp4 |
| --- | --- | --- | --- | --- |
| Trust in the government | 0.523 | -0.418 | 0.179 | -0.721 |
| Trust in the health care system | 0.488 | 0.322 | -0.811 | -0.034 |
| Trust in the parliament | 0.516 | -0.504 | 0.082 | 0.687 |
| Trust in the police | 0.471 | 0.684 | 0.551 | 0.082 |

Table CT2: Principal components of the trust in institutions index. The first component is considered sufficient, as the scree plot of eigenvalues in Fig CF2 shows.

Fig CF2: Scree plot of eigenvalues after principal component analysis for the trust in institutions index.

Social norms (IV)

As in the above cases, a PCA shows that one component is sufficient for combining the underlying variables of descriptive and injunctive norms (see Fig CF3). The single norm items cover the same aspects of preventive behaviour as in the dependent variable: *staying at home except for necessities*, *keeping distance of at least 1 meter*, and *wearing a mask whenever distance cannot be maintained*. The first component once again loads all underlying variables fairly equally (see Table CT3), and the correlation coefficient with the additive index is 0.75.

|  | Comp1 | Comp2 | Comp3 | Comp4 | Comp5 | Comp6 |
| --- | --- | --- | --- | --- | --- | --- |
| Descriptive norm: staying home | 0.421 | -0.531 | 0.075 | 0.292 | -0.276 | -0.611 |
| Descriptive norm: wearing mask | 0.383 | 0.464 | 0.476 | 0.442 | 0.459 | -0.077 |
| Descriptive norm: keeping distance | 0.415 | 0.104 | -0.537 | 0.475 | -0.271 | 0.479 |
| Injunctive norm: staying home | 0.406 | -0.570 | 0.208 | -0.281 | 0.377 | 0.496 |
| Injunctive norm: wearing mask | 0.418 | 0.337 | 0.359 | -0.464 | -0.600 | 0.088 |
| Injunctive norm: keeping distance | 0.406 | 0.231 | -0.555 | -0.447 | 0.371 | -0.370 |

Table CT3: Principal components of the social norms index. The first component is considered sufficient, as the scree plot of eigenvalues in Fig CF3 shows.

Fig CF3: Scree plot of eigenvalues after principal component analysis for the social norms index.

Health risks (IV)

According to the PCA, one component is sufficient for representing the two underlying variables *perceived personal health risk* and *perceived public health risk* (see Fig CF4). The first component loads both variables equally (see Table CT4). The correlation coefficient with the additive index is 0.91.

|  | Comp1 | Comp2 |
| --- | --- | --- |
| Perceived health risk: for the public | 0.707 | 0.707 |
| Perceived health risk: for oneself | 0.707 | -0.707 |

Table CT4: Principal components of the perceived health risks index. The first component is considered sufficient, as the scree plot of eigenvalues in Fig CF4 shows.

Fig CF4: Scree plot of eigenvalues after principal component analysis for the perceived health risks index.

Controls

Next to the dependent and independent variables described above, we use individuals’ perceived effectiveness of governmental measures as a control variable. Furthermore, we control for the sociodemographic characteristics of gender, age, education, household size, migration background, and employment status. Employment status is also included in the FE-regressions (see Table 1 in the main text and Appendix A2 and A3) and contains a dummy for flexible work arrangements (home office), which has been argued to affect possibilities for social distancing (Papageorge et al., 2020). We recoded all variables in a way that aligns the direction and range of the scales (normalization), thus easing comparability. Hence, every variable ranges from 0 to 1, whereby 0 indicates the lowest and 1 the highest value of the corresponding concept (i.e. frequency, trust, agreement to statements, estimations of opinions and behaviour). The exact wording for all questions, the corresponding answer options in German, and their translation into English can be found in Appendix C2. We provide basic descriptive statistics for all variables used in the analyses in Appendix A1.

### C2. Questions and answer options

Question wordings for all variables in the ACPP, in English and the German original, can also be found online (Kittel et al. 2020b).

DV: index preventive behaviour

In the following, think of **your personal behaviour** in the last week. Please specify **how often** you have **engaged in the following behaviour**: (randomized answer items)

1. You stay at home, except for necessary trips.
2. In public, you keep a minimum distance of 1m from people who do not live in your household.
3. In public, you always wear protective masks.

Answer options:

*1 = Almost always*

*2 = Most of the time*

*3 = Sometimes*

*4 = Rarely*

*5 = Almost never*

*don’t know [88]*

*no answer [99]*

Denken Sie im Folgenden an **Ihr persönliches** **Verhalten** in der letzten Woche. Bitte geben Sie an, **wie oft** Sie sich **wie folgt verhalten** **haben**: (randomisierte Antwort-Items)

1. Sie bleiben zu Hause, außer für Notwendigkeiten.
2. Sie halten im öffentlichen Raum immer mindestens 1m Abstand von Menschen, die nicht mit Ihnen im Haushalt leben.
3. Sie tragen immer Schutzmasken, wenn Sie sich im öffentlichen Raum bewegen.

Matrix-Labels:

*1 = Nahezu immer*

*2 = Meistens*

*3 = Manchmal*

*4 = Selten*

*5 = Nahezu nie*

*weiß nicht [88]*

*keine Angabe [99]*

IV: index social norms

*Injunctive norms*

In the following, think of the **opinions of other people in Austria**. Please specify **how many** Austrians **hold the following opinions**. There is no right or wrong answer here, it is about your personal estimation. (randomized answer items)

1. Everybody has to stay at home, except for necessary trips.
2. In public, everybody has to keep a minimum distance of 1m from people who do not live in their household.
3. In public, everybody must wear protective masks.

Answer options:

*1 = Almost everybody takes that view*

*2 = Most people take that view*

*3 = Approximately half of the people take that view*

*4 = Some people take that view*

*5 = Almost nobody takes that view*

*don’t know [88]*

*no answer [99]*

Denken Sie im Folgenden an die **Meinungen anderer Menschen in Österreich**. Bitte geben Sie an, **wie viele** Österreicherinnen und Österreicher die **folgenden Meinungen vertreten**. Es gibt hier kein richtig oder falsch, es geht nur um Ihre persönliche Schätzung. (randomisierte Antwort-Items)

1. Alle müssen unbedingt zu Hause bleiben, außer für Notwendigkeiten.
2. Alle müssen im öffentlichen Raum unbedingt immer mindestens 1m Abstand von Menschen halten, die nicht mit ihnen im Haushalt leben.
3. Alle müssen unbedingt Schutzmasken tragen, wenn sie sich im öffentlichen Raum bewegen.

Matrix-Labels:

*1 = Nahezu alle sind dieser Meinung*

*2 = Die meisten sind dieser Meinung*

*3 = Etwa die Hälfte ist dieser Meinung*

*4 = Einige sind dieser Meinung*

*5 = Nahezu niemand ist dieser Meinung*

*weiß nicht [88]*

*keine Angabe [99]*

*Descriptive norms*

In the following, don’t think of the opinions, but of the **actual behaviour of other people in Austria** instead. From your perspective, please specify **how many** Austrians **engage in the following behaviour**. There is no right or wrong answer here, it is about your personal estimation. (randomized answer items)

1. They stay at home, except for necessary trips.
2. In public, they keep a minimum distance of 1m from people who do not live in their household.
3. In public, they always wear protective masks.

Answer options:

*1 = Almost everybody behaves like this*

*2 = Most people behave like this*

*3 = Approximately half of the people behave like this*

*4 = Some people behave like this*

*5 = Almost nobody behaves like this*

*don’t know [88]*

*no answer [99]*

Denken Sie im Folgenden nicht mehr an die Meinung, sondern an das **tatsächliche Verhalten anderer Menschen in Österreich**. Bitte geben Sie an, **wie viele** Österreicherinnen und Österreicher sich Ihrer Meinung nach **wie folgt verhalten**. Es gibt hier kein richtig oder falsch, es geht nur um Ihre persönliche Schätzung. (randomisierte Antwort-Items)

1. Sie bleiben zu Hause, außer für Notwendigkeiten.
2. Sie halten im öffentlichen Raum immer mindestens 1m Abstand von Menschen, die nicht mit ihnen im Haushalt leben.
3. Sie tragen immer Schutzmasken, wenn sie sich im öffentlichen Raum bewegen.

Matrix-Labels:

*1 = Nahezu alle verhalten sich so*

*2 = Die meisten verhalten sich so*

*3 = Etwa die Hälfte verhält sich so*

*4 = Einige verhalten sich so*

*5 = Nahezu niemand verhält sich so*

*weiß nicht [88]*

*keine Angabe [99]*

IV: index trust in institutions

Please look at the following list: **In regards to the Corona crisis**, do you have a lot of, some, little, or no **trust** in the corresponding **institutions**? (randomized answer items)

1. The police
2. The parliament
3. The health care system
4. The federal government

Answer options:

*0 = No trust at all*

*1*

*2*

*3*

*4*

*5*

*6*

*7*

*8*

*9*

*10 = A lot of trust*

*don’t know [88]*

*no answer [99]*

Wenn Sie auf die folgende Liste sehen: Haben Sie sehr viel, ziemlich viel, wenig oder überhaupt kein **Vertrauen** in die jeweils genannten **Institutionen im Rahmen der Coronakrise**? (randomisierte Antwort-Items)

1. Die Polizei
2. Das Parlament
3. Das Gesundheitswesen
4. Die Bundesregierung

Matrix-Labels:

*0 = Überhaupt kein Vertrauen*

*1*

*2*

*3*

*4*

*5*

*6*

*7*

*8*

*9*

*10 = Sehr viel Vertrauen*

*weiß nicht [88]*

*keine Angabe [99]*

IV: index risk perception

**How high** do you estimate the **health threat** that the **coronavirus** poses for you personally and the Austrian population to be?

1. for **me** personally
2. for the **Austrian population**

Answer options:

*1 = very high*

*2 = high*

*3 = moderate*

*4 = low*

*5 = very low*

*no answer [99]*

**Wie groß** schätzen Sie die **gesundheitliche Gefahr** ein, welche von dem **Coronavirus** für Sie persönlich und für die österreichische Bevölkerung ausgeht?

1. für **mich** persönlich
2. für die **österreichische Bevölkerung**

Answer options:

*1 = sehr groß*

*2 = groß*

*3 = mittelmäßig*

*4 = klein*

*5 = sehr klein*

*keine Angabe [99]*

CV: effectiveness of measures

**How effective**, do you think, have been the **measures enacted by the Austrian government to slow the spread of the disease**?

1 = not effective at all

2 = rather ineffective

3 = partly effective

4 = rather effective

5 = very effective

don’t know [88]

no answer [99]

**Wie effektiv**, denken Sie, sind die gesetzten **Maßnahmen der österreichischen Regierung** bis jetzt, um die **Ausbreitung der Krankheit zu verlangsamen**?

1 = überhaupt nicht effektiv

2 = eher nicht effektiv

3 = teils effektiv

4 = eher effektiv

5 = sehr effektiv

weiß nicht [88]

keine Angabe [99]

## S1 Appendix D. Data and Code Availability

### D1. Data Availability

This study is based on data from the Austrian Corona Panel Project (ACPP, see Kittel et al., 2020a, 2020b). The first 20 waves of data have been published and can be downloaded after agreeing to sharing restrictions and ethics guidelines here: <https://doi.org/10.11587/28KQNS>

The ACPP processes requests to access pre-release editions of the dataset for academic purposes only. This dataset also includes wave 22 used in this study. A guide to apply for access can be found here: <https://viecer.univie.ac.at/coronapanel/austrian-corona-panel-data/access-request/>

Data on weekly COVID-19 cases in Austria can be accessed here: <https://info.gesundheitsministerium.at/data/Epikurve.csv>.

We are not authorized to share the data on individual cases per week by region but scientific institutions can apply for data access here: <https://datenplattform-covid.goeg.at/english>.

### D2. Code Availability

The R code (used to recode the data and produce the descriptive Figs) as well as the STATA code (used for the regressions and the interaction plots) to reproduce all our analyses and tests described in this manuscript can be accessed online at <https://osf.io/5pz26/?view_only=954cef37d8e84dfebce9278b7e2971cb>.

## S1 Appendix References

Angrist, J.D., Pischke, J.-S., 2008. Mostly harmless econometrics: an empiricist’s companion. Princeton University Press, Princeton.

Google, 2020. COVID-19 Community Mobility Report [WWW Document]. COVID-19 Community Mobility Report. URL https://www.google.com/covid19/mobility?hl=de (accessed 04.03.2021).

Hainmueller, J., Mummolo, J., Xu, Y., 2019. How Much Should We Trust Estimates from Multiplicative Interaction Models? Simple Tools to Improve Empirical Practice. Political Analysis 27, 163–192. https://doi.org/10.1017/pan.2018.46

Heiler, G., Reisch, T., Hurt, J., Forghani, M., Omani, A., Hanbury, A., Karimipour, F., 2020. Country-wide mobility changes observed using mobile phone data during COVID-19 pandemic. arXiv:2008.10064 [cs, stat].

Kittel, B., Kritzinger, S., Boomgaarden, H., Prainsack, B., Eberl, J.-M., Kalleitner, F., Lebernegg, N.S., Partheymüller, J., Plescia, C., Schiestl, D.W., Schlogl, L., 2020a. The Austrian Corona Panel Project: monitoring individual and societal dynamics amidst the COVID-19 crisis. Eur Polit Sci. https://doi.org/10.1057/s41304-020-00294-7

Kittel, B., Kritzinger, S., Boomgaarden, H., Prainsack, B., Eberl, J.-M., Kalleitner, F., Lebernegg, N.S., Partheymüller, J., Plescia, C., Schiestl, D.W., Schlogl, L., 2020b. Austrian Corona Panel Project (SUF edition). https://doi.org/10.11587/28KQNS

Papageorge, N.W., Zahn, M.V., Belot, M., Broek-Altenburg, E. van den, Choi, S., Jamison, J.C., Tripodi, E., 2020. Socio-Demographic Factors Associated with Self-Protecting Behavior during the Covid-19 Pandemic (No. w27378). National Bureau of Economic Research. https://doi.org/10.3386/w27378

1. We thank Heiler et al. for providing the data. For more details see their paper. [↑](#footnote-ref-1)
